# Use of Steroid Profiling Combined With Machine Learning for Identification and Subtype Classification in Primary Aldosteronism

**DOI:** 10.1001/jamanetworkopen.2020.16209

**Published:** 2020-09-29

**Authors:** Graeme Eisenhofer, Claudio Durán, Carlo Vittorio Cannistraci, Mirko Peitzsch, Tracy Ann Williams, Anna Riester, Jacopo Burrello, Fabrizio Buffolo, Aleksander Prejbisz, Felix Beuschlein, Andrzej Januszewicz, Paolo Mulatero, Jacques W. M. Lenders, Martin Reincke

**Affiliations:** 1 Department of Internal Medicine III, University Hospital Carl Gustav Carus, Technische Universität Dresden, Dresden, Germany; 2 Institute of Clinical Chemistry and Laboratory Medicine, University Hospital Carl Gustav Carus, Technische Universität Dresden, Dresden, Germany; 3 Biomedical Cybernetics Group, Biotechnology Center, Center for Molecular and Cellular Bioengineering, Center for Systems Biology Dresden, Department of Physics, Technische Universität Dresden, Dresden, Germany; 4 Center for Complex Network Intelligence Laboratory at the Tsinghua Laboratory of Brain and Intelligence, Department of Bioengineering, Tsinghua University, Beijing, China; 5 Division of Internal Medicine and Hypertension, Department of Medical Sciences, University of Turin, Turin, Italy; 6 Medizinische Klinik und Poliklinik IV, Klinikum der Ludwig-Maximilians-Universität München, Munich, Germany; 7 Department of Hypertension, Institute of Cardiology, Warsaw, Poland; 8 Department of Endocrinology, Diabetology, and Clinical Nutrition, UniversitätsSpital Zürich, Zürich, Switzerland; 9 Department of Internal Medicine, Radboud University Medical Center, Nijmegen, the Netherlands

## Abstract

**Importance:**

Most patients with primary aldosteronism, a major cause of secondary hypertension, are not identified or appropriately treated because of difficulties in diagnosis and subtype classification. Applications of artificial intelligence combined with mass spectrometry–based steroid profiling could address this problem.

**Objective:**

To assess whether plasma steroid profiling combined with machine learning might facilitate diagnosis and treatment stratification of primary aldosteronism, particularly for patients with unilateral adenomas due to pathogenic *KCNJ5* sequence variants.

**Design, Setting, and Participants:**

This diagnostic study was conducted at multiple tertiary care referral centers. Steroid profiles were measured from June 2013 to March 2017 in 462 patients tested for primary aldosteronism and 201 patients with hypertension. Data analyses were performed from September 2018 to August 2019.

**Main Outcomes and Measures:**

The aldosterone to renin ratio and saline infusion tests were used to diagnose primary aldosteronism. Subtyping was done by adrenal venous sampling and follow-up of patients who underwent adrenalectomy. Statistical tests and machine-learning algorithms were applied to plasma steroid profiles. Areas under receiver operating characteristic curves, sensitivity, specificity, and other diagnostic performance measures were calculated.

**Results:**

Primary aldosteronism was confirmed in 273 patients (165 men [60%]; mean [SD] age, 51 [10] years), including 134 with bilateral disease and 139 with unilateral adenomas (58 with and 81 without somatic *KCNJ5* sequence variants). Plasma steroid profiles varied according to disease subtype and were particularly distinctive in patients with adenomas due to *KCNJ5* variants, who showed better rates of biochemical cure after adrenalectomy than other patients. Among patients tested for primary aldosteronism, a selection of 8 steroids in combination with the aldosterone to renin ratio showed improved effectiveness for diagnosis over either strategy alone. In contrast, the steroid profile alone showed superior performance over the aldosterone to renin ratio for identifying unilateral disease, particularly adenomas due to *KCNJ5* variants. Among 632 patients included in the analysis, machine learning–designed combinatorial marker profiles of 7 steroids alone both predicted primary aldosteronism in 1 step and subtyped patients with unilateral adenomas due to *KCNJ5* variants at diagnostic sensitivities of 69% (95% CI, 68%-71%) and 85% (95% CI, 81%-88%), respectively, and at specificities of 94% (95% CI, 93%-94%) and 97% (95% CI, 97%-98%), respectively. The validation series yielded comparable diagnostic performance.

**Conclusions and Relevance:**

Machine learning–designed combinatorial plasma steroid profiles may facilitate both screening for primary aldosteronism and identification of patients with unilateral adenomas due to pathogenic *KCNJ5* variants, who are most likely to show benefit from surgical intervention.

## Key Points

**Question:**

Does steroid profiling combined with machine learning offer a potential 1-step strategy to facilitate diagnosis and subtype classification for treatment stratification of patients with primary aldosteronism?

**Findings:**

This diagnostic study involving patients tested for primary aldosteronism found that those with unilateral adenomas harboring pathogenic *KCNJ5* sequence variants showed the most clinical benefit from surgical intervention and could be effectively identified at a single screening step using machine-learning combinatorial marker profiles of 7 steroids.

**Meaning:**

The outlined strategy offers a potential approach to improve diagnosis of primary aldosteronism and facilitate more efficient and effective stratification of patients for surgical intervention.

## Teaser

This diagnostic study assesses whether plasma steroid profiling combined with machine learning facilitates diagnosis and treatment stratification of patients with primary aldosteronism, particularly those with unilateral adenomas due to pathogenic *KCNJ5* sequence variants.

## Introduction

Applications of artificial intelligence, including machine learning, are gaining increasing recognition for informing medical decision-making.^1,2,3,4^ Machine learning may be particularly useful in heterogeneous disorders where there is a need for stratification to guide therapy.^5,6,7,8^ One such disorder is primary aldosteronism (PA), a common cause of secondary hypertension with 2 main subtypes for which treatment stratification is crucial but difficult.^9,10^ With a prevalence of 5% to 7% among unselected patients with hypertension and up to 20% among patients with severe hypertension, PA affects large numbers of patients and is associated with considerable morbidity exceeding that of patients with primary hypertension (PHT) and similar elevations of blood pressure.^11,12^

The aforementioned considerations highlight the importance of effective methods for diagnosis and treatment of PA, which must allow for stratification according to unilateral vs bilateral hypersecretion of aldosterone.^9,10^ Cure of the former can be achieved by adrenalectomy, whereas mineralocorticoid receptor antagonists are indicated for the bilateral subtype. Attaining this stratification is not simple and usually requires adrenal venous sampling (AVS), a technically demanding, expensive, time-consuming, and not infallible procedure.^9,13,14,15,16^ In 2 independent studies,^13,16^ discordant lateralization results were observed in 24% to 28% of patients who underwent AVS with vs without adrenocorticotropin. In another study,^14^ clinical outcomes did not differ according to determination of unilateral disease by AVS vs radiological imaging. In a fourth study,^15^ there were no significant differences in rates of biochemical cure (76% vs 69%) in patients younger than 65 years who underwent adrenalectomy according to AVS lateralization ratios larger vs smaller than 4.

Apart from the difficulties and limited effectiveness of AVS for subtype classification, there are also problems with earlier steps in the diagnosis of PA. Although the aldosterone to renin ratio (ARR) offers a time-honored method for screening, there is considerable overlap of ratios among patients with and without PA^17,18^; thus, at ARR cutoffs selected to optimize diagnostic sensitivity, there are many false-positives, leading to the need for confirmatory studies.^19,20^ Such multiple steps, poor standardization, requirements to consider antihypertensive medications, and difficulties with AVS all represent barriers to diagnostic stratification; consequently, most patients remain undiagnosed and are not appropriately treated.^9,21^ Improved approaches for diagnostic stratification are therefore needed.

With the aforementioned considerations in mind, we examined the use of mass spectrometry–based steroid profiling combined with machine learning for diagnostic stratification, with the hypothesis that this approach at screening might facilitate case detection and also allow for subtype classification. This hypothesis was based on findings that distinct steroid profiles in adrenal venous plasma of patients with bilateral and unilateral PA translated to similarly distinct profiles in peripheral plasma.^22^ Patients with unilateral aldosterone-producing adenomas (APAs) due to pathogenic sequence variants of *KCNJ5* have particularly distinct steroid profiles.^23^ These patients also have larger and more clearly visualized APAs and show the most favorable outcomes after adrenalectomy.^24,25,26,27^ The use of steroid profiles to identify these patients may, therefore, be especially useful. Thus, the primary objective of this study was to establish whether steroid profiling could facilitate both identification and subtype classification of patients with PA, particularly those with unilateral APAs due to *KCNJ5* sequence variants.

## Methods

### Patients

This diagnostic study was approved by the Klinikum der Ludwig-Maximilians-Universität München, University of Turin, Technische Universität Dresden, and Institute of Cardiology (Warsaw). The study follows the Standards for Reporting of Diagnostic Accuracy (STARD) reporting guideline. All patients provided written informed consent under protocols approved by ethics committees at the 4 tertiary clinical care centers where patients were referred for testing.

The study involved mass spectrometry–based steroid profiling of plasma specimens from 462 patients tested for primary aldosteronism between June 13, 2013, and March 8, 2017. Follow-up of patients was completed by July 31, 2018. Patient data and specimens were derived from studies and registries with a focus on hypertension and PA, including the Conn registry in Germany, the European Network for Studies of Adrenal Tumors registry, and the Prospective Monoamine-Producing Tumor study.

Patients were tested for PA according to 1 or more of several criteria: office blood pressure greater than 150/100 mm Hg, therapy-resistant hypertension, or hypertension associated with hypokalemia or hemorrhagic stroke, an adrenal incidentaloma, or obstructive sleep apnea. Other forms of secondary hypertension were excluded when relevant. Testing for PA followed standard practice guidelines,^28^ including use of the ARR, confirmatory testing, and AVS to distinguish unilateral from bilateral PA (eAppendix 1 in the Supplement).

PA was confirmed in 304 patients included into the study according to selective sampling of both adrenal veins or in whom adrenalectomies were performed without AVS because of young age and imaging evidence of a single adrenal adenoma (eFigure 1 in the Supplement). Among these patients, 116 were defined by AVS to have bilateral disease. After exclusions among the others with unilateral disease, there remained 157 patients for whom Sanger sequencing for somatic variants of *KCNJ5* was performed in resected tumor specimens (eAppendix 1 in the Supplement). Two and 16 of the respective 60 and 97 patients with and without *KCNJ5* sequence variants did not experience complete biochemical cure and were reassigned as having bilateral PA according to the primary aldosteronism surgical outcome (PASO) classification system.^29^ PA was excluded in 158 patients, who were subsequently defined to have PHT. A further 201 patients with hypertension from a reference population^30^ were included to enhance patient numbers for generation of machine-learning algorithms (eTable 1 in the Supplement).

### Steroid Profiling

Steroid profiling was performed using liquid chromatography with tandem mass spectrometry,^31^ with details outlined in eAppendix 1 and eTable 2 in the Supplement. Measurements included 15 adrenal steroids: aldosterone, 18-oxocortisol, 18-hydroxycortisol, cortisol, cortisone, 11-deoxycortisol, 21-deoxycortisol, corticosterone, 11-deoxycorticosterone, progesterone, 17-hydroxyprogesterone, pregnenolone, androstenedione, dehydroepiandrosterone, and dehydroepiandrosterone-sulfate. Reference intervals were established as discussed elsewhere (eTable 3 in the Supplement).^30^

### Statistical Analysis

Statistical analyses used JMP Pro statistical software version 14 (SAS Institute). Unless otherwise specified, significance was defined as *P* < .05. Statistical tests were 2-tailed and included the Fisher exact test and the Mann-Whitney *U* test. Nominal logistic modeling was used to assess for associations of the presence versus absence of a pathogenic *KCNJ5* sequence variant with PASO criteria based–outcomes according to sex and age as additional covariates. Associations are shown according to whole model and likelihood ratio tests. Data for steroids were normalized by logarithmic transformation before analyses, including for generation of geometric means and 95% CIs. Least-squares multivariable models were used to assess differences in plasma steroids according to patient group, age, sex, and assay batch. Differences among patient groups were assessed using the Tukey honest significance test. Logistic regression was used to generate receiver operating characteristic curves, with selections of steroids in profiles based on both stepwise regression and likelihood ratios for each steroid. Differences between areas under receiver operating characteristic curves (AUROCs) and data from confusion matrices were used to assess performance of logistic regression models. Data were normalized according to upper cutoffs of reference intervals, which for most of the plasma steroids were specific for either or both age and sex (eTable 3 in the Supplement). Data analyses were performed from September 2018 to August 2019.

### Machine Learning

In brief, the machine-learning workflow involved 3 phases (eFigure 2 in the Supplement): data preparation, model learning, and external validation. Data preparation included several procedures for normalization, batch correction, and, in some models, adjustments for age and sex (see eTable 3, eTable 4, and eTable 5 in the Supplement). At this stage, each of the 13 different data sets was subdivided into 2 different proportions for learning and external validation data sets, as outlined in eAppendix 1 in the Supplement. After data preparation, machine-learning tasks for feature selection, model training, and sample classification in the second model learning phase were performed according to different algorithms, with their application in this phase restricted to learning data sets. Feature selection involved the use of 4 different algorithms to identify specific steroid combinations that provided either optimal segregation of patients with and without PA or identification of those with unilateral disease due to *KCNJ5* sequence variants among all patients.

Several combinations of the aforementioned procedures were investigated for optimized data analysis and assessed according to 9 machine-learning algorithms corresponding to variations of 4 commonly used models in medicine: random forest (RF), support vector machine (SVM), linear discriminant analysis, and logistic regression. A total of 585 models arising from 13 data sets and 9 machine-learning algorithms were tested, each involving a 10 times, 5-fold cross-validation step (eFigure 3 in the Supplement). Optimal classification, determined as part of the final validation phase according to either AUROC or *F* scores, was determined according to external validations achieved by application of algorithms for each of the 585 models applied to external validation data sets.

## Results

### Final Study Population

PA was confirmed in 273 patients (165 men [60%]; mean [SD] age, 51 [10] years). In addition to the 201 patients of the reference hypertension population, after screening and subtype classification, there were 158 patients classified with PHT (134 with bilateral PA and 139 with unilateral PA) (eTable 1 in the Supplement). Among those with unilateral PA, 58 had APAs due to *KCNJ5* variants and 81 did not and were designated as having wild-type *KCNJ5*.

### Genotype-Related Therapeutic Outcomes and Patient Group Reclassification

Among patients who underwent adrenalectomy because of presumed unilateral PA, those with APAs due to *KCNJ5 *variants were, on average, 5.8 years younger (mean [SD] age, 47.4 [10.8] years vs 51.3 [10.3] years) and were 2.7-fold more likely to be female (47 women [78.3%] vs 28 women [28.9%]) compared with those with wild-type *KCNJ5* APAs (Table 1). According to the PASO classification, the presence of *KCNJ5* variants conferred significantly better clinical and biochemical outcomes after adrenalectomy compared with the absence of *KCNJ5* variants. However, logistic modeling indicated that improved blood pressure control in patients with APAs due to *KCNJ5* variants vs wild-type APAs was accounted for by the younger age and female predominance of patients with *KCNJ5 *variants. In contrast, the presence of a *KCNJ5* variant remained independently associated with biochemical cure. The overall postadrenalectomy biochemical cure rate in this study was 88.5%; the cure rates were 96.6% for patients with* KCNJ5* variants and 83.6% for patients without *KCNJ5* variants.

**Table 1.** Comparisons of Age, Sex, and Primary Aldosteronism Surgical Outcome Clinical and Biochemical Outcomes in Patients With Adrenal Venous Sampling–Lateralized Evidence of Unilateral Adrenal Aldosterone Secretion According to the Presence or Absence of *KCNJ5* Sequence Variants in Resected Adenomas

Characteristic	Patients, No. (%)		*P* value
	Wild-type *KCNJ5 *(n = 97)	*KCNJ5 v*ariant (n = 60)	
Age, mean (SD), y	53.1 (10.3)	47.4 (10.8)	.002
Sex			
Female	28 (28.9)	47 (78.3)	<.001
Male	69 (71.1)	13 (27.7)	
Clinical outcomes of primary aldosteronism surgery^a^			
Complete cure	20 (20.6)	24 (40.0)	.008
Partial cure	52 (53.6)	30 (50.0)	
Failure	25 (25.8)	6 (10.0)	
Biochemical outcomes of primary aldosteronism surgery^b^			
Complete cure	81 (83.6)	58 (96.6)	.04
Partial cure	7 (7.2)	1 (1.7)	
Failure	9 (9.2)	1 (1.7)	

^a^ In the multivariate analyses for clinical outcomes, likelihood ratios were 9.34 for age impact (*P* = .009), 6.01 for sex impact (*P* = .05), and 1.42 for *KCNJ5* impact (*P* = .49), with *P* < .001 for the whole model.

^b^ In the multivariate analyses for biochemical outcomes, likelihood ratios were 9.15 for age impact (*P* = .01), 0.34 for sex impact (*P* = .85), and 7.16 for *KCNJ5* impact (*P* = .03), with *P* = .01 for the whole model.

### Steroid Profiles

With least squares adjustments of sex, age, and assay batch, all plasma steroids showed some differences among the 5 patient groups (eTable 4 in the Supplement). Plasma 18-oxocortisol showed differences among all groups but especially the group with unilateral APAs due to *KCNJ5* variants, in whom plasma concentrations were 6.2- to 10.3-fold higher than all other groups (Table 2). Plasma 18-hydroxycortisol in the *KCNJ5* variant group was also 3.3- to 4.0-fold higher than in other groups. Plasma aldosterone in the 2 unilateral disease groups, which did not differ, were higher than in the other 3 groups. Other steroids were either similarly increased in patients with PA or showed differing patterns or decreases or increases compared with patients with hypertension according to the particular subtype of PA.

**Table 2.** Plasma Concentrations of Steroids in Reference Patients With Hypertension, Patients With Primary Hypertension, and Patients with Bilateral Primary Aldosteronism or Unilateral Primary Aldosteronism Without and With *KCNJ5* Sequence Variants

Steroid	Plasma concentration, least square geometric mean (95% CI), nmol/L^a^				
	Hypertension		Primary aldosteronism		
	Reference	Primary	Bilateral	Unilateral with wild-type *KCNJ5*	Unilateral with *KCNJ5 *variant
Aldosterone	0.091 (0.077-0.106)	0.143 (0.119-0.169)	0.260 (0.222-0.302)	0.384 (0.312-0.463)	0.436 (0.341-0.543)
18-Oxocortisol	0.026 (0.022-0.031)	0.043 (0.035-0.052)	0.056 (0.047-0.066)	0.093 (0.074-0.114)	0.578 (0.440-0.735)
18-Hydroxycortisol	1.62 (1.41-1.84)	1.74 (1.50-1.99)	1.75 (1.54-1.97)	2.11 (1.79-2.46)	6.960 (5.71-8.34)
Corticosterone	4.28 (3.60-5.01)	5.50 (4.57-6.53)	7.21 (6.13-8.39)	6.36 (5.15-7.70)	7.11 (5.52-8.90)
11-Deoxycorticosterone	0.063 (0.052-0.075)	0.112 (0.091-0.135)	0.162 (0.136-0.191)	0.277 (0.220-0.342)	0.311 (0.235-0.397)
11-Deoxycortisol	1.332 (1.074-1.618)	1.610 (1.275-1.985)	2.935 (2.397-3.529)	4.500 (3.455-5.686)	2.917 (2.124-3.838)
21-Deoxycortisol	0.039 (0.030-0.050)	0.044 (0.032-0.057)	0.078 (0.060-0.099)	0.083 (0.059-0.111)	0.085 (0.056-0.120)
Cortisol	237 (213-262)	332 (296-370)	327 (296-360)	248 (218-280)	274 (235-317)
Cortisone	47.2 (43.2-51.4)	53.2 (48.3-58.4)	48.4 (44.5-52.5)	35.5 (31.8-39.4)	43.4 (38.2-49.1)
Androstenedione	2.47 (2.25-2.70)	2.69 (2.43-2.96)	3.48 (3.19-3.79)	2.87 (2.56-3.19)	3.16 (2.76-3.58)
Dehydroepiandrosterone	8.70 (7.66-9.81)	7.91 (6.89-8.99)	7.90 (7.01-8.85)	6.19 (5.30-7.15)	7.97 (6.63-9.45)
Dehydroepiandrosterone-sulfate	3401 (3061-3758)	2805 (2504-3125)	2718 (2461-2987)	2234 (1965-2520)	2506 (2151-2888)
17-Hydroxyprogesterone	1.10 (0.96-1.24)	1.39 (1.20-1.59)	2.07 (1.83-2.33)	1.95 (1.65-2.27)	2.05 (1.68-2.45)
Progesterone	0.336 (0.269-0.411)	0.277 (0.217-0.344)	0.595 (0.483-0.720)	0.577 (0.438-0.735)	0.431 (0.310-0.572)
Pregnenolone	1.95 (1.61-2.32)	2.12 (1.73-2.56)	1.51 (1.26-1.78)	2.09 (1.66-2.58)	2.30 (1.74-2.94)

^a^ Geometric means and 95% CIs were derived from the exponents of logarithmically transformed data. For whole model differences, see eTable 4 in the Supplement.

### Diagnostic Test Performance of the ARR and Plasma Steroids

**Figure 1.**
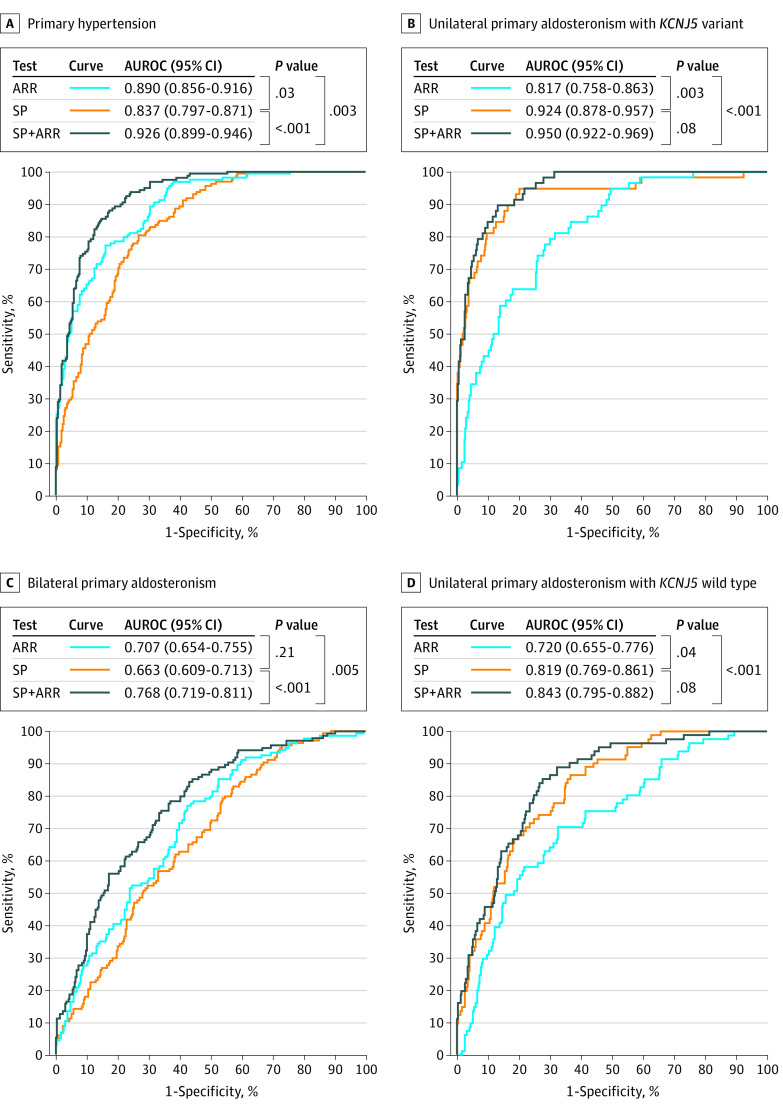
Areas Under the Receiver Operating Characteristic Curves (AUROCs) Comparing the Aldosterone to Renin Ratio (ARR) With a Steroid Profile (SP) and the Combination of the SP and the ARR Each of the 4 panels represents a comparison of AUROCs for the single indicated patient group with the other 3 groups combined. Thus, AUROCs for primary hypertension illustrate the diagnostic performance for distinguishing all patients with primary aldosteronism from primary hypertension, but with sensitivity illustrative for detection of primary hypertension. The 8 steroids included in the profile were aldosterone, 18-oxocortisol, 18-hydroxycortisol, 11-deoxycorticosterone, cortisol, cortisone, androstenedione, and dehydroepiandrosterone.

From differences in AUROCs, a selected panel of 8 steroids was less effective than the ARR (difference in AUROC, 0.053; 95% CI, 0.006 to 0.099; *P* = .03) for differentiating patients with PA from those with hypertension, but more effective (difference in AUROC, 0.107; 95% CI, 0.037 to 0.176; *P* = .003) for distinguishing patients with unilateral APAs due to pathogenic *KCNJ5 *variants from others (Figure 1). Combination of the steroid profile with the ARR was nevertheless more effective for discriminating PA from PHT than use of either the steroid profile (difference in AUROC, 0.089; 95% CI, 0.059 to 0.119; *P* < .001) or the ARR (difference in AUROC, 0.036; 95% CI, 0.013 to 0.060; *P* = .003) alone. Combination of the steroid profile with the ARR improved performance over the ARR alone for distinguishing patients with APAs due to *KCNJ5* variants from other patients (difference in AUROC, 0.134; 95% CI, 0.082 to 0.186; *P* < .001), but not the steroid profile alone (difference in AUROC, 0.027; 95% CI, −0.004 to 0.016; *P* = .08). Similar results were also observed for the unilateral APA group with wild-type *KCNJ5*, but for both this group and the bilateral PA group, all AUROCs were lower than for PHT and *KCNJ5* variant groups.

From confusion matrices, steroid profiles correctly identified nearly 3 times more patients with APAs due to *KCNJ5 *variants than the ARR (eTable 6 in the Supplement). With the addition of the steroid profile to the ARR, the diagnostic yield of patients correctly identified with PA increased from 69.6% (95% CI, 64.2%-75.0%) to 81.3% (95% CI, 76.7%-85.9%) at respective diagnostic specificities of 89.2% (95% CI, 84.3%-94.1%) and 89.9% (95% CI, 85.2%-94.6%).

### Steroid Profiling With Machine Learning

After batch corrections (eFigure 4 and eFigure 5 in the Supplement) and using feature selection within machine-learning approaches, combinatorial markers composed of up to 7 steroids were identified that offered best performance for discriminating patient groups (eFigure 5, eFigure 6, eFigure 7, eFigure 8, eFigure 9, and eFigure 10 in the Supplement). Among those steroids, aldosterone, 18-oxocortisol, and 18-hydroxycortisol commonly occupied the top 3 places for discriminatory power. The next steroid with useful discriminatory power was 11-deoxycorticosterone, followed by several others depending on the model.

**Figure 2.**
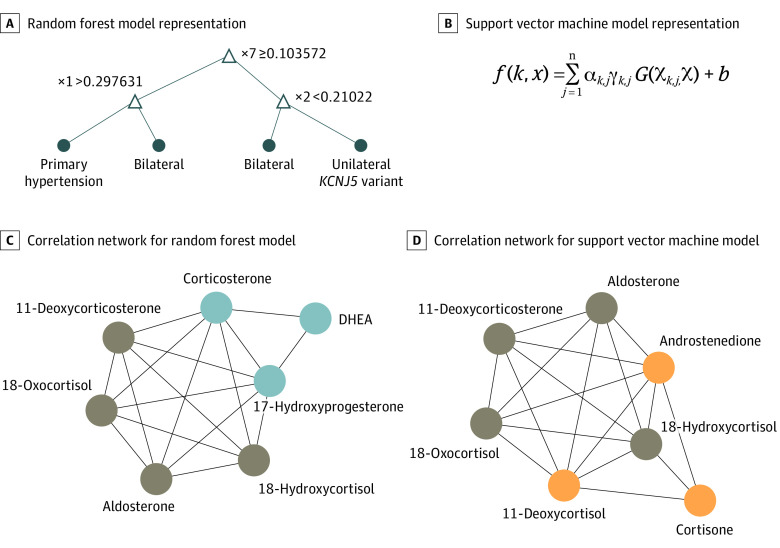
Results for the 2 Best Machine-Learning Models Panels A and C show a random forest (RF) model for the differentiation of primary hypertension (HT) from primary aldosteronism. Panels B and D show a support vector machine (SVM) with a nonlinear kernel model for the differentiation of patients with unilateral aldosterone-producing adenomas due to *KCNJ5* sequence variants in primary aldosteronism vs other groups. Panel A shows the subtree from 1 decision tree of 500 in the model representing how random forest predicts new samples. Panel B outlines the mathematical formula used in SVM to predict new sample scores, where *k* is the number of binary SVM models created for the 1 vs 1 approach for multiclass SVM training, *x* is the new sample to be predicted, *n* is the number of support vectors for the *k*th binary SVM, α and *b* are the parameters learned from the training step of the *k*th binary SVM, γ is the class of the respective *k*th support vector (1 or −1), and *G*(Χ*_k,j_*,Χ) is the dot product between the *j*th support vector hyperplane measures in the binary SVM *k* with the (new) sample measurements *x*. Correlation networks from the respective selected features for each model are shown in panels C and D, with nodes in brown showing common features. DHEA indicates dehydroepiandrosterone.

The final selection of models for optimal classification was reduced to 21 best models according to either AUROCs or *F* scores (eTable 7 in the Supplement). Among these, an RF model provided optimal performance for the classification of patients with and without PA, whereas a nonlinear (radial basis function kernel) SVM model was optimal for patients with APAs due to *KCNJ5 *variants (Figure 2). For both models, aldosterone, 18-oxocortisol, and 18-hydroxycortisol occupied the top 3 places, with 11-deoxycorticosterone following in fourth and fifth places, respectively, for the SVM and RF models. For the SVM model, cortisone, 11-deoxycortisol, and androstenedione replaced corticosterone, 17-hydroxyprogesterone, and dehydroepiandrosterone as selected features of the RF model.

Performance of RF and SVM models upon external validation was similar or even appeared to exceed that of the learning series (Table 3), according to 10 cross-validations in 5-folds (eAppendix 2 and eFigure 2 in the Supplement). Comparisons of AUROCs and *F* scores indicated that the SVM model performed nearly as well as the RF model for identifying patients with PA, but was consistently better for identifying those with APAs due to *KCNJ5* variants. Using the RF model to identify patients with PA, among all 632 patients, diagnostic sensitivity in the learning series was 69% (95% CI, 68%-71%) and specificity was 94% (95% CI, 93%-94%). The external validation series yielded sensitivity of 85% and specificity of 100%. To identify patients with APAs due to *KCNJ5* variants, the SVM model yielded a diagnostic sensitivity of 85% (95% CI, 81%-88%) at a specificity of 97% (95% CI, 97%-98%). The external validation series yielded respective values of sensitivity of 100% and specificity of 98%.

**Table 3.** Confusion Matrices and Diagnostic Performance for the 2 Machine-Learning Models (RF-Gini and SVMnl-RFE) for the Learning (Training and Testing) and External Validation Series of Patients With PHT, B-PA, and Unilateral Primary Aldosteronism With and Without *KCNJ5* Sequence Variants

Actual groups	Predicted groups							
	Learning^a^				External validation^b^			
	PHT	B-PA	Wild-type *KCNJ5*	*KCNJ5* variant	PHT	B-PA	Wild-type *KCNJ5*	*KCNJ5 v*ariant
RF-Gini								
Confusion matrices								
PHT	60.4	2.4	1.2	0.6	36	0	0	0
B-PA	9	11	2.9	1.2	3	6	3	1
Wild-type *KCNJ5*	4.1	5.4	3.4	1.7	1	4	3	0
*KCNJ5 v*ariant	2	1.7	2	4.6	0	0	1	5
Diagnostic performance^c^								
Sensitivity, %	94 (93-94)	42 (40-45)	26 (24-29)	46 (42-49)	100	46	38	83
Specificity, %	69 (68-71)	89 (89-90)	94 (94-95)	97 (96-97)	85	92	93	98
AUROC	0.815 (0.807-0.825)	0.657 (0.645-0.670)	0.599 (0.585-0.613)	0.714 (0.690-0.730)	0.926	0.691	0.651	0.908
PPV, %	80 (79-81)	52 (50-55)	38 (32-40)	59 (55-64)	90	60	43	83
NPV, %	89 (88-90)	86 (85-87)	90 (89-91)	95 (94-95)	100	87	91	98
*F* score	0.863 (0.858-0.870)	0.464 (0.440-0.480)	0.309 (0.279-0.336)	0.516 (0.484-0.548)	0.947	0.522	0.400	0.833
SVMnl-RFEl								
Confusion matrices								
PHT	63.2	1.2	0.2	0	35	1	0	0
B-PA	10.6	10.8	1.6	1.2	4	5	4	0
Wild-type *KCNJ5*	2.8	3.6	6.6	1.6	2	1	4	1
*KCNJ5 *variant	1.2	0.2	0.2	8.8	0	0	0	6
Diagnostic performance^c^								
Sensitivity, %	98 (97-98)	45 (43-47)	45 (42-49)	85 (81-88)	97	38	50	100
Specificity, %	70 (69-73)	94 (94-95)	98 (88-98)	97 (96-97)	78	96	93	98
AUROC	0.841 (0.831-0.850)	0.695 (0.684-0.706)	0.716 (0.700-0.736)	0.909 (0.890-0.920)	0.875	0.672	0.714	0.991
PPV, %	81 (80-83)	69 (67-71)	78 (74-81)	77 (74-80)	85	71	50	86
NPV, %	95 (94-95)	86 (85-87)	92 (91-93)	98 (97-99)	95	86	93	100
*F* score	0.888 (0.883-0.895)	0.537 (0.516-0.556)	0.562 (0.528-0.593)	0.801 (0.777-0.825)	0.909	0.500	0.500	0.923

Abbreviations: AUROC, area under the receiver operating characteristic curve; B-PA, bilateral primary aldosteronism; NPV, negative predictive value; PHT, primary hypertension; PPV, positive predictive value.

^a^ For learning series, numbers in confusion matrices reflect 5-folds of patients (ie, 569/5 = 114 patient for each fold) with evaluations of each fold performed 10 times within each learning series (thus, numbers represent the mean of 50 confusion matrices).

^b^ For the external validation series numbers reflect the learning proportions (90:10) and 10% (63) of the total number of patients (632) in the analysis.

^c^ Values for diagnostic performance in learning series are shown with 95% CI, whereas those for validation series are not.

As outlined in eAppendix 2 in the Supplement, the aforementioned measures of diagnostic performance were derived using learning ratios of 90% optimal for training and testing (thus, 10% for external validation), which was particularly important for the limited population of 58 patients with APAs due to *KCNJ5* variants. Thus, measures of diagnostic performance for the SVM model of both learning and external validation series, but particularly the latter, showed improvement as the learning ratio increased from 50% to 90% (eFigure 11 in the Supplement). For the RF model, for which population sizes of PA and hypertensive groups were both relatively large, measures of diagnostic performance showed little difference between learning and validation series until the learning ratio reached 90% (eFigure 12 in the Supplement).

## Discussion

To our knowledge, this study is the first to demonstrate the application of multidimensional pattern recognition and machine learning for analysis of steroidomic data in the diagnosis of PA. This approach offers the potential for more efficient and effective diagnostic stratification than the traditional series of multiple studies involving single end-point measures in relation to given cutoff values. Stratification was achieved by distinct steroid profiles among subgroups of patients with PA. From those profiles, panels of steroids were identified that can facilitate diagnosis of PA and, in the same screening step, identify patients with APAs due to *KCNJ5 *variants for triaging as candidates likely to show beneficial therapeutic outcomes from further interventions.

Among the steroids in the panel with distinctive profiles, 18-oxocortisol and 18-hydroxycortisol stood out from the others for identifying patients with unilateral APAs due to *KCNJ5* variants and were consistently among the top 4 steroids selected by machine-learning algorithms. Previous studies have identified those hybrid steroids to be produced in excess in some patients with PA,^32,33,34,35,36^ but only recently has it been clarified that elevations of these steroids are linked to APAs with pathogenic variants of *KCNJ5*.^23,37,38^ The 2 hybrid steroids appear to be formed by actions of aldosterone synthase on 11-deoxycortisol,^39^ which is normally produced in the zona fasciculata and converted there to cortisol by 11β-hydroxylase. Production of the hybrid steroids by APAs due to *KCNJ5* variants is explained by their zona fasciculata phenotype and their expression of both *CYP11B1* and *CYP11B2*.^40^

The benefits of AVS over imaging to establish unilateral PA are well established.^41^ Nevertheless, the originally suggested high diagnostic accuracy of AVS for indicating unilateral disease^42^ has not been supported by some subsequent studies involving postadrenalectomy follow-up.^14,15^ In the present study, the 88.5% postadrenalectomy biochemical cure rate lies between those found previously^29,41,43^ and is similar to that found in a single prospective study.^14^ The failure of adrenalectomy to cure PA may reflect asymmetric bilateral disease in some patients.^44^ Aldosterone-producing cell clusters have been identified in the zona glomerulosa of aging adrenal glands and in the adrenal glands of patients with PA due to bilateral adrenal aldosterone hypersecretion; in both cases, cells of those clusters are characterized by high rates of pathogenic variants of *CACNAID*, but not *KCNJ5.*^45,46^ This raises the possibility that *KCNJ5* sequence variants might be characteristic of unilateral adenomas. Nevertheless, 2 of our patients with APAs due to *KCNJ5* variants did not experience complete biochemical cure after adrenalectomy, suggesting that *KCNJ5* sequence variants are not strictly associated with unilateral disease. Nevertheless, failure to reach cure in patients with APAs due to *KCNJ5* variants was rare, confirming findings that these patients show more clinical benefit after adrenalectomy than others.^25,26,27^ As we further establish here, the benefit in terms of biochemical cure is independent of age and sex, further highlighting the importance of triaging patients with APAs due to *KCNJ5* variants for further interventions.

There have been other studies that combined steroid profiling with machine learning,^47,48^ but, to our knowledge, this is the first to apply a combinatorial marker design strategy to PA. The potential benefits for diagnostic stratification of PA are multiple. First, during screening it may be possible to more effectively distinguish patients with PA from those with other causes of hypertension. Second, by identifying within the same screening step patients with unilateral APAs due to *KCNJ5* variants, it should be possible to immediately triage those patients for AVS; alternatively, with clear imaging evidence of a unilateral adenoma, it may be possible to directly proceed to an adrenalectomy without AVS. These considerations underscore the potential advantages of moving away from traditional unidimensional approaches (eg, ARR) for diagnostic stratification to multidimensional approaches that take advantage of today’s computational power for applications of artificial intelligence.

### Limitations

As detailed in eAppendix 3 in the Supplement, the present analysis has limitations that are typical of retrospective diagnostic studies, including dependence on traditional methods to establish patient classifications. It thus cannot be guaranteed that PA was excluded in all patients designated as having primary hypertension or that some cases of bilateral PA were incorrectly classified. Although patient follow-up ensured that final cases of unilateral PA were correctly classified, reliance on Sanger sequencing for identifying *KCNJ5* variants is not 100% sensitive; it is, thus, possible that some wild-type *KCNJ5* cases may have been incorrectly classified. Measurements of the ARR at sampling time points different from those used for steroid profiling, batch effects, and inconsistencies in supine and seated blood sampling represent other limitations. Seated sampling, which increases plasma renin and aldosterone, likely accounts for the higher levels of aldosterone and 18-oxocortisol in patients with PHT who were screened for PA compared with those of the reference hypertensive population, for whom sampling was performed with patients in the supine position. As outlined in the eAppendix 3 in the Supplement, because sampling for steroid profiles among patients with PA was mainly performed with patients in the supine position, this may have adversely impacted the performance of steroid profiles for distinguishing patients with PA from those with PHT.

## Conclusions

These findings suggest that plasma steroid profiles obtained during initial screening for PA can improve case detection beyond that possible using the ARR alone. Moreover, the use of distinctive profiles to identify patients with unilateral APAs due to *KCNJ5* variants further illustrates the potential of steroid profiling for disease stratification at a single screening step. Along with advances in functional imaging^49,50,51^ and other measurements, such as the angiotensin peptidome,^52,53^ steroid profiling combined with machine learning may facilitate more rapid identification of patients with PA for appropriate therapeutic interventions. As detailed in eAppendix 3 in the Supplement, such strategies are now being tested in further patient populations, and with those developments it may become possible to screen more than the small proportion of patients with PA who are currently tested and treated according to disease subtype.

## Notes

Supplement. **eAppendix 1.** Supplemental Methods **eFigure 1.** SPISCA Study Patient Flow Diagram **eTable 1. **Demographic Data for the Five Final Groups of the Study Population **eTable 2.** Interassay Coefficients of Variation (CV) for Three Different Quality Control Materials Containing the Steroids at Differing Plasma Concentrations (ng/mL) **eTable 3. **Upper Cutoffs of Reference Intervals for Plasma Steroids **eFigure 2. **Workflow for the Analysis of the Plasma Steroidomic Data by Machine Learning (ML) According to Three Steps **eTable 4. **Multivariate Analysis Using a Model That Takes Into Account Sex, Age and Assay Batch to Establish Differences Among the Five Patient Groups: 1. Reference Hypertensives; 2. Primary Hypertensives; 3. Bilateral PA; 4. Unilateral PA Without *KCNJ5* Mutations; and 5 Unilateral PA With *KCNJ5* Mutations **eTable 5**. Normalizations and Methods for the 54 Selected Top Machine Learning Models **eAppendix 2. **Supplemental Results **eTable 6.** Confusion Matrices and Diagnostic Performance From Logistic Regression Analyses of Aldosterone:Renin Ratios (ARR), Steroid Profiles and Combined ARR and Steroid Profiles for Patients With Primary Hypertension (PHT) or Primary Aldosteronism (PA) According to Bilateral Disease or Unilateral *KCNJ5* Wildtype (KCNJ5^WT^) or Mutation-Positive (KCNJ5^MUT^) Disease **eFigure 3.** AUC for the Assessment of Batch Influence and Correction for Each Steroid **eFigure 4. **Outputs Forest Plots by the Tool exploBATCH Quantifying the Batch Effects **eFigure 5. **Listings of Models With Selected Features and Bar Graphs of Diagnostic Performance (External Validation Series) for the First Set of Nine ML Algorithms According to Criteria 1 and Evaluations by AUC **eFigure 6. **Listings of Models With Selected Features and Bar Graphs of Diagnostic Performance (External Validation Series) for the Second Set of Nine ML Algorithms According to Criteria 2 and Evaluations by AUC **eFigure 7. **Listings of Models With Selected Features and Bar Graphs of Diagnostic Performance (External Validation Series) for the Third Set of Nine ML Algorithms According to Criteria 3 and Evaluations by AUC **eFigure 8. **Listings of Models With Selected Features and Bar Graphs of Diagnostic Performance (External Validation Series) for the Fourth Set of Nine ML Algorithms According to Criteria 3 and Evaluations by F-Score **eFigure 9. **Listings of Models With Selected Features and Bar Graphs of Diagnostic Performance (External Validation Series) for the Fifth Set of Nine ML Algorithms According to Criteria 2 and Evaluations by F-Score **eFigure 10. **Listings of Models With Selected Features and Bar Graphs of Diagnostic Performance (External Validation Series) for the Sixth Set of Nine ML Algorithms According to Criteria 2 and Evaluations by F-Score **eTable 7. **Final 21 Unique Top Performing ML Models **eFigure 11. **Measures of Diagnostic Performance for Identification of Patients With *KCNJ5* Mutation+ve APAs Using the SVMnl-RFE Model **eAppendix 3. **Supplemental Discussion **eFigure 12. **Measures of Diagnostic Performance for Identification of Patients With Primary Hypertension Using the RF-Gini Model **eReferences**
