# Supplementary material for: Use of Steroid Profiling Combined With Machine Learning for Identification and Subtype Classification in Primary Aldosteronism
Source: JAMA Netw Open. 2020 Sep 29;3(9):e2016209. doi: 10.1001/jamanetworkopen.2020.16209 (PMC7525346; doi:10.1001/jamanetworkopen.2020.16209)
Supplement: Supplement. — eAppendix 1. Supplemental Methods eFigure 1. SPISCA Study Patient Flow Diagram eTable 1. Demographic Data for the Five Final Groups of the Study Population eTable 2. Interassay Coefficients of Variation (CV) for Three Different Quality Control Materials Containing the Steroids at Differing Plasma Concentrations (ng/mL) eTable 3. Upper Cutoffs of Reference Intervals for Plasma Steroids eFigure 2. Workflow for the Analysis of the Plasma Steroidomic Data by Machine Learning (ML) According to Three Steps eTable 4. Multivariate Analysis Using a Model That Takes Into Account Sex, Age and Assay Batch to Establish Differences Among the Five Patient Groups: 1. Reference Hypertensives; 2. Primary Hypertensives; 3. Bilateral PA; 4. Unilateral PA Without KCNJ5 Mutations; and 5 Unilateral PA With KCNJ5 Mutations eTable 5. Normalizations and Methods for the 54 Selected Top Machine Learning Models eAppendix 2. Supplemental Results eTable 6. Confusion Matrices and Diagnostic Performance From Logistic Regression Analyses of Aldosterone:Renin Ratios (ARR), Steroid Profiles and Combined ARR and Steroid Profiles for Patients With Primary Hypertension (PHT) or Primary Aldosteronism (PA) According to Bilateral Disease or Unilateral KCNJ5 Wildtype (KCNJ5WT) or Mutation-Positive (KCNJ5MUT) Disease eFigure 3. AUC for the Assessment of Batch Influence and Correction for Each Steroid eFigure 4. Outputs Forest Plots by the Tool exploBATCH Quantifying the Batch Effects eFigure 5. Listings of Models With Selected Features and Bar Graphs of Diagnostic Performance (External Validation Series) for the First Set of Nine ML Algorithms According to Criteria 1 and Evaluations by AUC eFigure 6. Listings of Models With Selected Features and Bar Graphs of Diagnostic Performance (External Validation Series) for the Second Set of Nine ML Algorithms According to Criteria 2 and Evaluations by AUC eFigure 7. Listings of Models With Selected Features and Bar Graphs of Diagnostic Performance (External Validation [file jamanetwopen-e2016209-s001.pdf]

Supplementary Online Content

Eisenhofer G, Durán C, Cannistraci CV, et al. Use of steroid profiling combined with machine learning for identification and subtype classification in primary aldosteronism. *JAMA Netw Open*. 2020;3(9):e2016209. doi:10.1001/jamanetworkopen.2020.16209

eAppendix 1. Supplemental Methods
eFigure 1. SPISCA Study Patient Flow Diagram
eTable 1. Demographic Data for the Five Final Groups of the Study Population
eTable 2. Interassay Coefficients of Variation (CV) for Three Different Quality Control Materials Containing the Steroids at Differing Plasma Concentrations (ng/mL)
eTable 3. Upper Cutoffs of Reference Intervals for Plasma Steroids
eFigure 2. Workflow for the Analysis of the Plasma Steroidomic Data by Machine Learning (ML) According to Three Steps
eTable 4. Multivariate Analysis Using a Model That Takes Into Account Sex, Age and Assay Batch to Establish Differences Among the Five Patient Groups: 1. Reference Hypertensives; 2. Primary Hypertensives; 3. Bilateral PA; 4. Unilateral PA Without *KCNJ5* Mutations; and 5 Unilateral PA With *KCNJ5* Mutations
eTable 5. Normalizations and Methods for the 54 Selected Top Machine Learning Models
eAppendix 2. Supplemental Results
eTable 6. Confusion Matrices and Diagnostic Performance From Logistic Regression Analyses of Aldosterone:Renin Ratios (ARR), Steroid Profiles and Combined ARR and Steroid Profiles for Patients With Primary Hypertension (PHT) or Primary Aldosteronism (PA) According to Bilateral Disease or Unilateral *KCNJ5* Wildtype ($KCNJ5^{WT}$) or Mutation-Positive ($KCNJ5^{MUT}$) Disease
eFigure 3. AUC for the Assessment of Batch Influence and Correction for Each Steroid
eFigure 4. Outputs Forest Plots by the Tool exploBATCH Quantifying the Batch Effects
eFigure 5. Listings of Models With Selected Features and Bar Graphs of Diagnostic Performance (External Validation Series) for the First Set of Nine ML Algorithms According to Criteria 1 and Evaluations by AUC
eFigure 6. Listings of Models With Selected Features and Bar Graphs of Diagnostic Performance (External Validation Series) for the Second Set of Nine ML Algorithms According to Criteria 2 and Evaluations by AUC
eFigure 7. Listings of Models With Selected Features and Bar Graphs of Diagnostic Performance (External Validation Series) for the Third Set of Nine ML Algorithms According to Criteria 3 and Evaluations by AUC
eFigure 8. Listings of Models With Selected Features and Bar Graphs of Diagnostic Performance (External Validation Series) for the Fourth Set of Nine ML Algorithms According to Criteria 3 and Evaluations by F-Score
eFigure 9. Listings of Models With Selected Features and Bar Graphs of Diagnostic Performance (External Validation Series) for the Fifth Set of Nine ML Algorithms According to Criteria 2 and Evaluations by F-Score
eFigure 10. Listings of Models With Selected Features and Bar Graphs of Diagnostic Performance (External Validation Series) for the Sixth Set of Nine ML Algorithms According to Criteria 2 and Evaluations by F-Score

© 2020 Eisenhofer G et al. *JAMA Network Open.*

eTable 7. Final 21 Unique Top Performing ML Models
eFigure 11. Measures of Diagnostic Performance for Identification of Patients With *KCNJ5* Mutation+ve APAs Using the SVMnl-RFE Model
eAppendix 3. Supplemental Discussion
eFigure 12. Measures of Diagnostic Performance for Identification of Patients With Primary Hypertension Using the RF-Gini Model
eReferences

This supplementary material has been provided by the authors to give readers additional information about their work.

© 2020 Eisenhofer G et al. *JAMA Network Open.*

eAppendix 1. Supplemental Methods

Study objectives

The primary objective of the SPISCA study (**S**teroid **P**rofiling for **I**dentification and **S**ubtype **C**lassification of Primary **A**ldosteronism) was to assess the utility of peripheral venous plasma steroid profiling with machine learning (ML) for identification and subtype classification of patients with primary aldosteronism (PA). This objective was based on findings of distinct profiles of steroids in adrenal venous plasma among patients with different subtypes of PA.[1,2] Differences in adrenal venous steroid profiles were reflected by distinct fingerprints in peripheral venous plasma, which might minimize requirements for adrenal venous sampling (AVS) in order to achieve subtype classification and stratification of patients for therapeutic intervention. Those earlier studies, however, focused on AVS, were thus restricted to patients with PA, and included patients receiving ACTH as part of AVS studies, which may compromise interpretation of peripheral plasma steroid profiles. The SPISCA study therefore included patients with primary hypertension and was restricted to patients with PA who had not received ACTH. Although steroid profiling alone was not predicted to accurately distinguish patients with PA from those with primary hypertension, it was predicted that steroid profiling within the larger screening population would facilitate identification of subsets of patients with primary aldosteronism, particularly those with aldosterone-producing adenomas (APAs) due to somatic mutations of *KCNJ5*.

Selection of patients into the study population

Patients included those with PA or hypertension (eFigure 1) who were enrolled under Ethics committee approved clinical protocols at four tertiary care referral centers: 1. Klinikum der Ludwig-Maximilians-Universität München, Munich, Germany; 2. Technische Universität Dresden, Dresden, Germany; 3. Department of Medical Sciences, University of Turin, Turin, Italy and 4. Institute of Cardiology, Warsaw, Poland. Selection of patients for testing of PA and the procedures used in their diagnostic work-up were based on Endocrine Society guidelines.[3] For those with PA and a diagnosis of unilateral disease, inclusion into the final study population depended on requirements of surgical intervention (i.e., adrenalectomy) and follow-up according to criteria detailed further below. Furthermore among patients with unilateral APAs, tumor tissue must have undergone testing for somatic mutations of *KCNJ5*.

For the diagnosis of unilateral versus bilateral PA based on AVS, all blood samplings were carried out without ACTH stimulation. Inclusion of patients into the study also required that AVS must have been selective for both adrenal veins according to a cortisol selectivity index of larger than 2.0. AVS-based diagnosis of unilateral PA required a lateralization ratio larger than 4.0 or alternatively larger than 3.0 when contralateral suppression was evident (<1.0 compared to a peripheral sample). To reach adequate numbers of patients required for formulation of ML algorithms, the study took advantage of data from peripheral venous samples of 144 patients with PA reported previously[1,2] and inclusion of a second set of data from 160 PA patients. After exclusions, 273 patients with PA were selected into the final study population (eFigure 1).

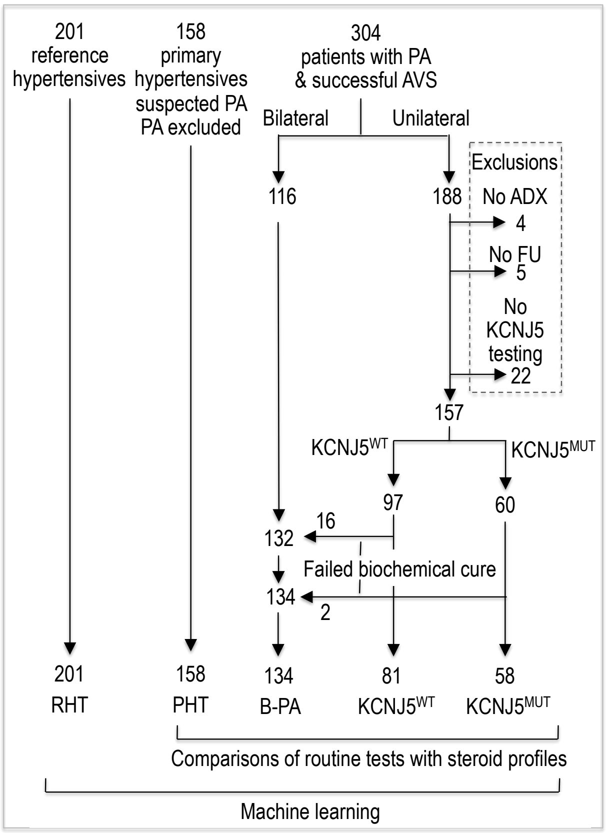

Supplemental figure 1. SPISCA study patient flow diagram. Abbreviations: PA, primary aldosteronism; AVS, adrenal venous sampling; ADX, adrenalectomy; FU, follow-up, RHT, reference hypertension; PHT, primary hypertension; B-PA, bilateral PA; KCNJ5^{WT}, wildtype; KCNJ5^{MUT}, mutant

The diagnosis of primary hypertension required that all secondary forms of hypertension had been excluded. In particular, among patients with hypertension and an elevated aldosterone:renin

© 2020 Eisenhofer G et al. *JAMA Network Open.*

ratio (ARR), PA was excluded using an intravenous or oral salt-loading test. Pheochromocytoma, Cushing syndrome, renal artery stenosis and obstructive sleep apnea syndrome were excluded according to standard clinical procedures in those patients in whom these diagnoses were suspected as determined by clinical clues derived from medical histories and physical examinations.

Patients in whom the aforementioned inclusion criteria could not be met were excluded from the final study population. Patients with serious or life threatening comorbidities (e.g., cancer, heart failure, renal failure etc) as well as other conditions that might impact adrenal function (e.g., adrenal insufficiency) were also excluded from participation in the study. In addition to the steroid profiling carried out in the above patient groups, the study population also included a group of 201 volunteers with hypertension (reference hypertensives) in whom steroid profiles were available as detailed elsewhere[4] and who were included to reach adequate numbers of patients for ML (eFigure 1).

Patient follow-up

Follow-up of patients with a diagnosis of unilateral PA and who underwent adrenalectomy was carried out to confirm the diagnosis according to the therapeutic response to the surgical intervention. Follow-up was performed between 6 and 12 months after adrenalectomy. Follow-up during that period included measurements of serum potassium, the ARR, saline suppression testing when the ARR returned positive results and documentation of blood pressure and antihypertensive medications (including doses), which were managed according to the blood pressure response to adrenalectomy. Comparisons of follow-up to screening blood pressure-related variables were according to recordings of these variables before any changes were made to antihypertensive medications for assessment of the ARR. Assessments of therapeutic response utilized the PA Surgical Outcome (PASO) classification system.[5]

The PASO classification system was developed based on consensus (Delphi method) among experts in the field on follow-up intervals and on criteria to define outcomes after adrenalectomy for unilateral PA. Outcome was defined separately for clinical and biochemical follow-up evaluation and this was based on blood pressure, use of antihypertensive drugs, plasma potassium and aldosterone concentrations, and plasma renin concentrations or activities. For defining final outcomes after adrenalectomy, consensus was reached that follow-up should be assessed between 6 and 12 months after surgery. For outcome assessment, consensus was reached on six possible outcome categories: complete clinical success, partial clinical success, absent clinical success, complete biochemical success, partial biochemical success and absent biochemical success.

Only patients who showed complete biochemical success after adenalectomy were finally assessed to have unilateral PA. In this way 18 of the 157 (11.5%) patients included in the final study population with initial evidence of unilateral PA were reassigned to the bilateral PA group (eFigure 1). The final five groups of patients were characterized by differences in sex and age that were particularly pronounced within the three groups of patients with PA and specially for the proportions of female to male sexes in patients with unilateral PA due to *KCNJ5* mutations ($KCNJ5^{MUT}$) compared to those without mutations ($KCNJ5^{WT}$) or with bilateral PA (eTable 1). The female predominance of patients with *KCNJ5* mutations in adenoma is a well established yet unexplained finding.

eTable 1. Demographic Data for the Five Final Groups of the Study Population

	Reference hypertension	Primary hypertension	Bilateral PA	Unilateral PA $KCNJ5^{WT}$	Unilateral PA $KCNJ5^{MUT}$
N	201	158	134	81	58
Sex (F/M)	89/112	77/81	40/94	22/59	46/12
% F	44.3±6.9	48.7±7.8	29.9±7.7*	27.2±9.7*	79.3±10.4**
Age (yr)	51.3±1.8*	47.2±2.4	51.2±1.8	53.0±2.2*	47.8±2.6†

Proportions of females (%F) and age as mean values are shown with 95% confidence intervals. Abbreviations: PA, primary aldosteronism; WT, wildtype. *P<0.05, compared to primary hypertension; **P<0.001 compared to all other patient groups; †P<0.05, compared to $KCNJ5^{MUT}$.

Blood samples
Peripheral venous blood samples (heparin or EDTA) for plasma steroid profiling were collected from all patients during morning hours (07:30-11:00). Most samples in patients with PA were taken in the supine position during AVS; however, samples for 19 patients were taken at screening in the seated position (i.e., 7% in seated and 93% in supine position). Almost all samples in the 158 patients with primary hypertension were taken in the seated position, whereas samples for the 201 hypertensive volunteers were all taken in the supine position as detailed elsewhere.[4] After centrifugation to separate blood cells, plasma specimens were stored at -70ºC or lower before shipping on dry ice to the Technische Universität Dresden for measurements of steroids by liquid chromatography with tandem mass spectrometry (LC-MS/MS).

LC-MS/MS-based steroid profiling
Measurements of steroids were originally based on an LC-MS/MS method initially applied to AVS applications, as detailed previously.[6] Although this method also allowed for measurements in peripheral venous plasma, this was not optimal for some steroids present at low abundance in peripheral samples (e.g., 18-oxocortisol). Therefore, the original method was further optimized as detailed below. Inter-assay coefficients of variation (CV) after method optimization remained under 15% for most steroids at the three different concentrations (89% of samples); exceptions included five steroids where CVs ranged between 15% and 20%, but this was usually at the lower concentrations (eTable 2).

eTable 2. Interassay Coefficients of Variation (CV)* for Three Different Quality Control Materials Containing the Steroids at Differing Plasma Concentrations (ng/mL)

Steroid	Conc	CV [%]	Conc	CV (%)	Conc	CV (%)
Aldosterone	0.044	17.4	0.18	9.4	3.88	8.3
18-Oxocortisol	0.351	10.8	1.46	15.6	3.45	14.3
18-Hydroxycortisol	0.773	15.5	3.42	8.7	3.67	9.9
Corticosterone	0.480	8.1	1.22	5.5	18.37	6.4
11-Deoxycorticosterone	0.285	7.5	1.16	6.3	11.53	9.7
11-Deoxycortisol	0.183	8.2	0.55	7.9	5.12	6.9
21-Deoxycortisol	0.228	7.8	0.94	5.3	4.71	5.0
Cortisol	36.72	10.6	71.89	6.8	70.71	6.0
Cortisone	1.138	7.3	4.69	6.4	5.36	5.5
Androstenedione	0.123	14.2	0.48	9.5	6.53	10.7
DHEA	0.549	20.0	3.85	9.2	4.85	11.9
DHEAS	29.14	12.8	109.40	8.1	45.69	12.9
Pregnenolone	n.d.	n.d.	2.09	19.8	5.67	11.1
Progesterone	0.260	11.0	1.08	8.7	4.99	8.1
17-Hydroxyprogesterone	0.381	4.9	1.22	4.8	22.51	4.2

*CVs were obtained from nine different assay runs during the time period that the second batch of SPISCA samples were measured by LC-MS/MS. Pregnenolone was not detectable (nd) in one of the three materials used for quality control. Abbreviations: Conc, concentration; n.d. not detectable..

Method optimization included change from use of atmospheric pressure chemical ionization to positive electrospray ionization for determination of 18-oxocortisol. Therefore, respective multiple reaction monitoring transitions were re-optimized and revealed product ion masses at quadrupole three of 313.1 and 77.0 as respective quantifier and qualifier ions, using m/z of 377.1 as the quadrupole 1 parent mass. Declustering and entrance potentials were optimized at 96eV and 10eV, respectively. Collision energies and cell exit potentials were optimized at 29eV and 22eV, and 127eV and 12eV for respective quantifier and qualifier ions. Furthermore, calibration ranges were optimized for the much lower concentrations in peripheral venous than adrenal venous plasma. Calibrator concentrations (n=6) ranged from 0.0125-5 ng/mL for aldosterone, 0.025-10 ng/mL for androstenedione and 11-deoxycortisol, 0.0625-25 ng/mL for

© 2020 Eisenhofer G et al. *JAMA Network Open.*

corticosterone, 11-deoxycorticosterone, DHEA, progesterone, 17-hydroxyprogesterone and 21-deoxycortisol, 0.125-50 ng/mL for 18-oxocortisol, 0.25-100 ng/mL 18-hydroxycortisol, 2.5-1,000 ng/mL for cortisol and 6.25-2,500 ng/mL for DHEAS. For the latter it became further necessary to separate those calibrators from all others, particularly from DHEA, due to small traces of DHEA as impurities in the DHEAS calibrator substance. For pregnenolone, a calibration range of 0.125-50 ng/mL was established, with revalidation studies indicating a lower limit of quantification at 2 ng/mL.

All plasma samples from patients with primary hypertension were analyzed after method optimization. For patients with PA, 66 (50%), 26 (20%) and 40 (30%) respective patients with bilateral disease and unilateral APAs with and without KCNJ5 mutation were analyzed before method optimization compared to 68 (48%), 32 (23%) and 41 (29%) after optimization.

Reference intervals for plasma steroids

Reference intervals for plasma concentrations of steroids were established after method optimization. The age- and sex-specific reference intervals for the 15 steroids employed in the present study have been described in detail elsewhere[4] and are available in an online open access data-in-brief zip file associated with that report.[4] That data-in-brief file contains the complete population dataset used for calculations of reference intervals and outlines the best-fit polynomial coefficients and associated variables used for curve-fitting and generation of equations for gender- and age-specific reference intervals, as required. The data-in-brief file also contains tables of references intervals and formulae for reference intervals as well as interactive nomograms that allow for calculation of age- and sex-specific reference intervals according to input of data. A simplified table containing the upper-cutoffs of reference intervals applied to the 15 steroids of the present study is also provided here (eTable 3) to assist with appreciation of the need to express or normalize plasma concentrations of steroids according to age and/or sex.

eTable 3. Upper Cutoffs of Reference Intervals for Plasma Steroids

Steroid	Males	Females
Aldosterone	0.45	0.67
18-Oxocortisol	0.10	0.09
18-Hydroxycortisol	4.64	3.40
Cortisol	644	700
11-Deoxycortisol	2.58	1.69
21-Deoxycortisol	0.45	0.22
Androstenedione*	$y = 60.82x^{-0.766} + 3.68$	$y = 13.92e^{-0.017x}$
DHEA*	$y = 93.71e^{-0.025x}$	$y = 84.49e^{-0.025x}$
DHEAS*	$y = -0.07x + 12.43$	$y = -0.002x^2 + 0.12x + 6.11$
Cortisone*	$y = 122.2e^{-0.007x}$	$y = 386.8x^{-0.43}$
Corticosterone*	$y = 546.6x^{-0.801}$	$y = 43120x^{-1.92}$
11-Deoxycorticosterone*	$y = 2.85x^{-0.533}$	$y = 0.65e^{-0.0122x}$
Pregnenolone*	y = 57.1e-0.028x	y = 1138x-1.188
17-Hydroxyprogesterone*†	$y = -0.08x + 9.42$	6.84 & 2.49
Progesterone†	0.7	2.5, 54.8

All upper-cut-offs are in nmol/L except for DHEAS, which is in µmol/L. *For androstenedione, DHEA, DHEA-S, cortisone, corticosterone, 11-deoxycorticosterone and 17-hydroxyprogesterone in males, upper cut-offs are shown by formulae that relate the cut-off value (y) to age (x). †For 17-hydroxyprogesterone and progesterone in females two cut-offs are shown for females before and after menopause respectively.

Sequencing for *KCNJ5* mutations

KCNJ5 genotyping was performed by Sanger sequencing of genomic DNA extracted from fresh frozen tumor nodules. CYP11B2 expression was confirmed by immunohistochemistry of formalin fixed paraffin

© 2020 Eisenhofer G et al. *JAMA Network Open.*

embedded tumors using specific CYP11B2 antibodies (a kind gift from Prof Celso Gomez-Sanchez University of Mississippi, USA).

Machine learning

The procedure for the supervised classification of the four subject groups was carried out according to a workflow involving three phases that included several trials of data preparation and use of different ML algorithms (eFigure 2).

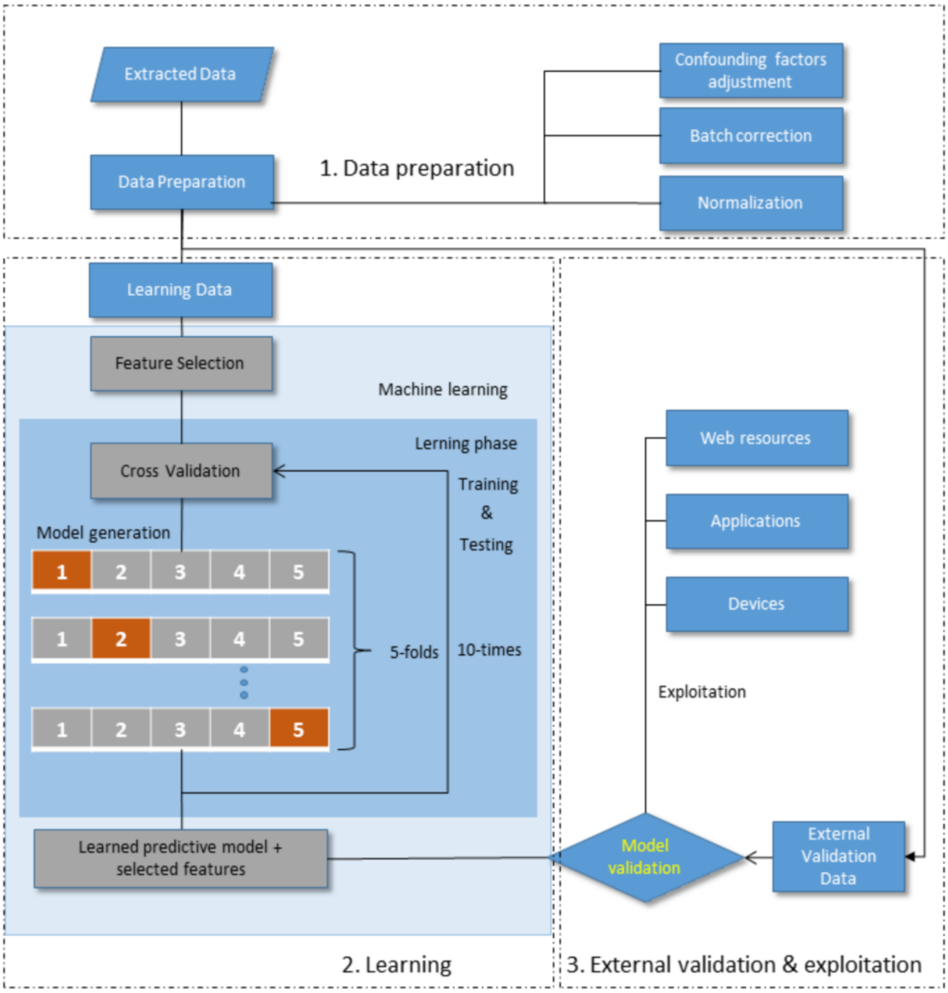

eFigure 2. Workflow for the Analysis of the Plasma Steroidomic Data by Machine Learning (ML) According to Three Steps.

1. data preparation, 2. training and testing the model in learning phase, and 3. external validation and exploitation. Selection of steroids most useful as predictors for classification (feature selection) was carried out before the ML learning phase. Cross-validation in the learning phase was applied to reduce over-fitting of the model. The trained predictive models were then provided together with the selected features, which were used for combinatorial markers. Finally, the models were evaluated by testing their performance on prediction of patient subgroups using an external (separate) set of data. Exploitation of the model is then possible (assuming performance thresholds are passed) according to new sources of data and applications.

As a consequence of method optimizations, some variable batch-to-batch differences became apparent for several steroids according to assays run before compared to after method optimization (eTable 4). For most steroids these impacts were relatively minor compared to group differences or impacts of age or sex. The major exception was pregnenolone, which showed particularly pronounced higher measured values after compared to before method optimization. Due to these differences and because of highly variable plasma concentrations of progesterone in premenopausal women, those two steroids were excluded from consideration in ML algorithms.

© 2020 Eisenhofer G et al. *JAMA Network Open.*

eTable 4. Multivariate Analysis Using a Model That Takes Into Account Sex, Age and Assay Batch to Establish Differences Among the Five Patient Groups: 1. Reference Hypertensives; 2. Primary Hypertensives; 3. Bilateral PA; 4. Unilateral PA Without *KCNJ5* Mutations; and 5 Unilateral PA With *KCNJ5* Mutations.

	Patient group		Sex		Age		Batch	
	Logworth*	P-value	Logworth	P-value	Logworth	P-value	Logworth	P-value
Aldosterone	34.81	<0.0001	2.99	0.0001	2.33	0.0028	2.17	0.0068
Nature of any difference			F>M		-ve relationship		1.38	
18-Oxocortisol	65.17	<0.0001	0.01	0.9759	1.39	0.0408	2.26	0.0055
Nature of any difference					-ve relationship		0.66	
18-Hydroxycortisol	32.25	<0.0001	4.49	<0.0001	0.56	0.2786	0.33	0.4720
Nature of any difference			M>F				0.92	
Corticosterone	4.22	<0.0001	0.45	0.3543	1.31	0.0492	1.63	0.0234
Nature of any difference					-ve relationship		1.34	
11-Deoxycorticosterone	28.51	<0.0001	0.11	0.7841	0.72	0.1891	0.46	0.3451
Nature of any difference							1.12	
11-Deoxycortisol	12.26	<0.0001	0.17	0.6774	0.41	0.3902	1.53	0.0296
Nature of any difference							1.29	
21-Deoxycortisol	3.93	0.0001	0.66	0.2174	0.29	0.5170	3.28	0.0005
Nature of any difference							0.55	
Cortisol	7.97	<0.0001	1.12	0.0764	0.18	0.6627	0.84	0.1428
Nature of any difference							1.13	
Cortisone	6.21	<0.0001	0.44	0.3609	1.90	0.0128	0.24	0.5770
Nature of any difference					-ve relationship		1.04	
Androstenedione	5.49	<0.0001	1.34	0.0461	26.19	<0.0001	12.22	<0.0001
Nature of any difference			M>F		-ve relationship		1.63	
DHEA	1.87	0.0136	1.15	0.0714	47.96	<0.0001	1.99	0.0102
Nature of any difference					-ve relationship		0.79	
DHEAS	5.48	<0.0001	20.64	<0.0001	47.45	<0.0001	0.67	0.2125
Nature of any difference			M>F		-ve relationship		1.09	
Pregnenolone	1.53	0.0296	0.32	0.4773	13.01	<0.0001	18.24	<0.0001
Nature of any difference					-ve relationship		3.99	
Progesterone	5.18	<0.0001	15.86	<0.0001	11.45	<0.0001	1.40	0.0403
Nature of any difference			F>M		-ve relationship		1.35	
17-Hydroxyprogesterone	11.48	<0.0001	40.21	<0.0001	9.06	<0.0001	2.39	0.0040
Nature of any difference			M>F		-ve relationship		1.30	

* Logworth is defined as the -log of the P-value (the higher the logworth the lower the P-value) and provides a transformation to compare model effects.. Nature of differences for model effects are shown for P-values < 0.05 (i.e., logworth values > 1.30). For differences related to group classifications see figure 1. The nature of batch one to two differences is shown as a ratio of batch two to one. For these values, data for patients with hypertension were omitted.

As part of the data preparation phase, data were normalized to generate several datasets according to six normalization methods (eTable 5): 1. use of the logarithm to the base 10 (LOG); 2. use of the square root (SQRT); 3. division of each cell-column (feature) by its column sum (DCS); 4. division of each cell-row (sample) by its row sum (DRS); 5. use of the Z-score; and 6. use of the logarithm to the base10 of the fold-difference of concentrations from an age and sex-specific reference population mean (LOG-FM). The latter normalization, combining logarithmic transformation with normalization according to sex and age, took advantage of a previously described reference population in which equations were derived by polynomial

© 2020 Eisenhofer G et al. *JAMA Network Open.*

and other regression models and thereby used to establish age- and sex-specific 95 percentiles and population means.[4] Data were also analyzed without normalization (no Norm).

Since the data were obtained from two LC-MS/MS assay batches (see LC-MS/MS-based steroid profiling), a batch correction procedure was also incorporated into the data preparation step (eTable 5).

eTable 5. Normalizations and Methods for the 54 Selected Top Machine Learning Models

Performance evaluation by AUC							Performance evaluation by F-score					
No	Criteria	Model	Norm method	Batch correction	TP		No	Criteria	Model	Norm method	Batch correction	TP
1	1	SVMl-RFE	DRS	Combat	0.9		28	1	SVMl-RFE	DRS	combat	0.9
2	1	SVMl-EN	LOG-FM	RD	0.7		29	1	SVMl-EN	SQRT	combat	0.9
3	1	SVMnl-RFE	LOG-FM	RD	0.9		30	1	SVMnl-RFE	LOG-FM	RD	0.9
4	1	SVMnl-EN	LOG-FM	RD	0.9		31	1	SVMnl-EN	DCS	PPCCA	0.9
5	1	RF-Gini	DRS	PPCCA	0.9		32	1	RF-Gini	DRS	PPCCA	0.9
6	1	RF-EN	LOG-FM	RD	0.9		33	1	RF-EN	SQRT	PPCCA	0.9
7	1	PLSDA	DCS	Combat	0.9		34	1	PLSDA	DCS	Combat	0.9
8	1	LDA-EN	LOG-FM	RD	0.9		35	1	LDA-EN	LOG-FM	RD	0.9
9	1	LR-EN	LOG-FM	RD	0.9		36	1	LR-EN	LOG-FM	RD	0.8
10	2	SVMl-RFE	DRS	combat	0.9		37	2	SVMl-RFE	DRS	combat	0.9
11	2	SVMl-EN	LOG-FM	RD	0.7		38	2	SVMl-EN	LOG-FM	RD	0.7
12	2	SVMnl-RFE	LOG-FM	RD	0.9		39	2	SVMnl-RFE	LOG-FM	RD	0.9
13	2	SVMnl-EN	LOG	PPCCA	0.9		40	2	SVMnl_EN	LOG	PPCCA	0.9
14	2	RF-Gini	DRS	PPCCA	0.9		41	2	RF-Gini	DRS	PPCCA	0.9
15	2	RF-EN	LOG	PPCCA	0.9		42	2	RF-EN	no Norm	PPCCA	0.9
16	2	PLSDA	DCS	Combat	0.9		43	2	PLSDA	DCS	Combat	0.9
17	2	LDA-EN	LOG-FM	RD	0.6		44	2	LDA-EN	LOG-FM	RD	0.6
18	2	LR-EN	LOG-FM	RD	0.7		45	2	LR-EN	LOG-FM	RD	0.7
19	3	SVMl-RFE	Z-score	combat	0.9		46	3	SVMl-RFE	DRS	combat	0.9
20	3	SVMl-EN	SQRT	combat	0.9		47	3	SVMl-EN	SQRT	combat	0.9
21	3	SVMnl-RFE	LOG-FM	RD	0.9		48	3	SVMnl-RFE	LOG-FM	RD	0.9
22	3	SVMnl_EN	LOG-FM	RD	0.9		49	3	SVMnl-EN	SQRT	combat	0.9
23	3	RF-Gini	DRS	PPCCA	0.9		50	3	RF-Gini	DRS	PPCCA	0.9
24	3	RF-EN	LOG-FM	RD	0.9		51	3	RF-EN	SQRT	PPCCA	0.9
25	3	PLSDA	DCS	Combat	0.9		52	3	PLSDA	DCS	Combat	0.9
26	3	LDA-EN	LOG-FM	RD	0.9		53	3	LDA-EN	LOG	PPCCA	0.9
27	3	LR-EN	LOG-FM	RD	0.9		54	3	LR-EN	LOG-FM	RD	0.8

Abbreviations: AUC, area under curve; Norm Method, normalization method; TP, training proportion; SVM, support vector machine; RF, random forest; PLSDA, partial least square discriminant analysis; LDA, linear discriminant analysis; LR, logistic regression; l, linear; nl, non-linear; RFE, recursive feature elimination; EN, elastic net; DRS, division of each cell-row by its row sum; LOG-FM, logarithm to the base10 of the fold-difference of concentrations from an age and sex-specific reference population mean; DCS, division of each cell-column by its column sum; RD, ratios of differences; PPCCA , probabilistic principal component and covariate analysis; no Norm, no normalization.

Two established algorithms for batch corrections were used: combating batch effect (Combat) and probabilistic principal component and covariates analysis (PPCCA) using an R tool called exploBATCH.[7] A third batch correction was also employed that used ratios of differences (RD) in steroids between batches according to least square multivariate analyses, accounting for group, sex and age. For this, corrections were applied exclusively to the first assay batch, which assumed assay optimization-associated improvements in accuracy of measurements in the second batch. Since samples assayed in the first batch did not include patients with primary hypertension, those patients were excluded from least squares multivariate analyses for this particular batch correction. According to that analysis, and as outlined in eTable 4 the largest batch-to-batch difference was observed for pregnenolone (3.99-fold higher in batch two than batch one, P<0.0001), which was excluded from subsequent ML analyses. Next highest presumed batch differences were observed for androstenedione, followed by 21-deoxycortisol, 18-oxocortisol, aldosterone, corticosterone, 17-hydroxyprogesterone, 11-deoxycortisol and DHEA. Other steroids showed no or negligible differences with ratios of batch two to one differences of between 0.92 to 1.13. For consistency, all steroids in batch one were adjusted downwards or upwards according to those ratios. This third batch correction was only applied to data normalized according to the LOG-FM method.

ML was initiated after data preparation (eFigure 2). Since the steroids in the panel were assumed to carry vital information for the segregation of the sample classes (i.e., hypertension, bilateral PA and unilateral PA with and without *KCNJ5* mutations), different feature selection algorithms were first applied according to the different ML procedures. Essentially this process facilitated the selection of crucial features (steroids) for the segregation of the sample classes.

Among employed feature selection algorithms, elastic net is a well-known algorithm that requires a parameter, alpha, which combines the L1 and L2 penalties of lasso and ridge regularization methods at different proportions.[8] Since the optimal alpha value was not known a priori, it was automatically tuned by changing its value from 0.1 until 0.9 in steps of 0.1. The alpha value that provided optimal performance was obtained by evaluating each elastic net output by means of areas under curves (AUC). Another method for feature selection that was employed, Gini index, is appropriate for random forest (RF) based algorithms.[9,10] This method determines what features are the most important to split sample classes, based on scores that depend on how many trees of the random forest are selected as split criteria. A further feature selection strategy, termed recursive feature elimination (RFE), was used for Support Vector Machine (SVM) algorithms.[11] That method with the help of an external estimator, in this case SVM, assigns weights to features to recursively prune them until a desired number of features is eventually reached. Since SVM does not support multiclass per se, a mean weight for each feature was obtained by averaging weights assigned by SVM between pairs of classes. The last feature selection strategy is intrinsically used for partial least square discriminant analysis (PLSDA) and was carried out by calculating the regression coefficients of partial least squares (PLS) and ranking them according to the number of latent variants for PLS.[12]

To minimize over-fitting for the feature selection steps, all algorithms were carried out ten times in a 10-fold cross validation procedure. Final numbers of features selected for each algorithm were defined by two steps: first, a fixed value (m) was computed by averaging the numbers of features obtained at each cross validation step and similarly the mean weight (w) of each feature was also computed across at each cross validation step; second, the features were sorted according to w and the m top features were selected for the respective models.

Once the predictors (features or specifically steroids) were chosen, various ML models were created in a 10 times 5-fold cross validation step (eFigure 2). The algorithms used for these ML models included SVM, RF, linear discriminant analysis (LDA), PLSDA and logistic regression (LR), but involved four variations of SVM models (SVMl-RFE, SVMl-EN, SVMnl-RFE, and SVMnl-EN) and two variations of RF models (RF-Gini and RF-EN). Variations for SVM models were based on use of RFE or elastic net (EN) for feature selection in combination with linear (SVMl) or non-linear radial basis function (SVMnl) kernels. The two variations of RF models reflected use of either the Gini index (Gini) or EN. Thus, a total of nine ML models were compared for the task of multiclass segregation (eTable 5).

The four SVM models were produced with the auto optimization of hyperparameters, which was also employed for the LDA model (with features selected from elastic net). The two RF models contained five hundred decision trees and were generated with several default parameters: fraction of input data to sample

with replacement 1; minimum number of observations per tree leaf 1; and number of variables to select at random for each decision split 3 corresponding approximately to the square root of number of variables, in this case 13. The PLSDA model was computed with default parameters (tolerant of convergence 1E-10), which was the same strategy employed for LR (nominal model and logit link function). Models were evaluated for performance in both learning (training and test) and external validation phases according to six measures: 1. sensitivity, 2. specificity, 3. areas under ROC curves (AUC), 4. positive predictive values, 5. negative predictive values and 6. F-scores.

Models were automatically selected according to three main criteria (eTable 5): 1. best performance for KCNJ5 mutation positive disease and PA versus primary hypertension; 2. best performance for all three PA groups vs primary hypertension; and 3. best performance for KCNJ5 mutation positive disease. Two measures were used for the selection of the models for each criteria: AUC and F-score (trade-off between sensitivity and positive predictive values). In practice, all the models were obtained following three principal steps:

1. Initial data segregation: The thirteen datasets (provided by different batch correctors and normalizations) were initially sub-divided into learning dataset and external validation dataset. The learning dataset was used to learn the "final models" using the above mentioned CV steps (which alternate by rotation the CV folds during training and testing; see also Supplemental figure 2), whereas the external validation dataset was used to assess the performance of "final models". The external validation dataset therefore is never 'provided' to the models during learning phase. For accuracy, we considered different initial segregations; the considered proportions (in percentages) of learning/external validation datasets were 50/50, 60/40, 70/30, 80/20 and 90/10.
2. Learning phase: A 10 times, repeated 5 folds CV was applied. This means that, for each time, the learning dataset is randomly divided into 5 folds. Then a training/test procedure is utilized: 4 folds are used together for training the models (80% of the data) and 1 for testing them (20% of the data). The average of the testing performances is considered to evaluate the model learning ability. Note that the evaluation of model learning performance is different from the evaluation of model performance on external validation data that is described at the third step below.
3. External validation phase: The models were further externally validated to predict the sample labels of the external validation data. This external validation works akin to emulate the event that a new patient is presenting (unknown class) to the clinician and we apply the markers associated to the model to predict its state. This provides an independent procedure to externallly test model performance since the external validation data were never used to train models. Since four classes are presented, the performance measures for each class were obtained by comparing the respective class (positive class) against all the rest (negative class).

eAppendix 2. Supplemental Results

Confusion matrices from logistic regression for comparisons of ARR and steroid profiles

Confusion matrices derived from LR comparisons of the ARR and steroid profiles indicated that steroid profiles showed more than 2-fold higher levels of diagnostic sensitivity than the ARR for identification of patients with both *KCNJ5* mutated APAs (55.2% vs 19.0%) and KCNJ5 wildtype APAs (37.0% vs 17.3%) at comparable specificities (97.1% vs 97.3% and 93.1% vs 95.1%). In contrast, sensitivity of the ARR for identification of patients with bilateral PA was superior to that of steroid profiles (53.0% vs 37.3%). Consequently from reversals of sensitivities and specificities for identification of patients with primary hypertension, steroid profiles and the ARR showed similar respective sensitivities (68.5% vs 69.6%) for identification of patients with PA, but at a higher specificity for the ARR than for steroid profiles (89.2% vs 80.4%). The combination of the ARR and steroid profiles led to improved diagnostic sensitivity (81.3%) at a similar level of specificity (89.9%) to the ARR (89.2%) for identification of patients with PA. Diagnostic sensitivities and specificities for identification the three PA subgroups also showed overall improvement with the combination of steroid profiles and the ARR. With that combination only one of the 58 KCNJ5^{MUT}cases was misclassified with primary hypertension and only one of the 158 patients with primary hypertension was misclassified as a KCNJ5^{MUT}case.

© 2020 Eisenhofer G et al. *JAMA Network Open.*

Importantly, the aforementioned results for diagnostic sensitivity and specificity for use of the ARR ias a screening test for PA were derived from analyses of ROC curves and do not reflect diagnostic performance according to upper cut-offs of reference intervals for the ARR, which were optimized to minimize false-negative results. At those cut-offs, diagnostic sensitivity of the ARR was 84.5% and specificity was 71.5%.

eTable 6. Confusion Matrices and Diagnostic Performance From Logistic Regression Analyses of Aldosterone:Renin Ratios (ARR), Steroid Profiles* and Combined ARR and Steroid Profiles for Patients With Primary Hypertension (PHT) or Primary Aldosteronism (PA) According to Bilateral Disease or Unilateral *KCNJ5* Wildtype (KCNJ5^{WT}) or Mutation-Positive (KCNJ5^{MUT}) Disease.

ARR		Predicted				Diagnostic performance†			
	Group	PHT	Bilateral PA	KCNJ5 WT	KCNJ5 MUT	Sensitivity		Specificity	
Actual	PHT	**141**	17	0	0	141/158	89.2%	190/273	69.6%
	Bilateral PA	55	**71**	5	3	71/134	53.0%	213/297	71.7%
	KCNJ5^{WT}	20	40	**14**	7	14/81	17.3%	333/350	95.1%
	KCNJ5^{MUT}	8	27	12	**11**	11/58	19.0%	363/373	97.3%
Steroid profile		Predicted				Diagnostic performance†			
	Group	PHT	Bilateral PA	KCNJ5 WT	KCNJ5 MUT	Sensitivity		Specificity	
Actual	PHT	**127**	29	1	1	127/158	80.4%	187/273	68.5%
	Bilateral PA	66	**50**	14	4	50/134	37.3%	226/297	76.1%
	KCNJ5^{WT}	14	31	**30**	6	30/81	37.0%	326/350	93.1%
	KCNJ5^{MUT}	6	11	9	**32**	32/58	55.2%	362/373	97.1%
ARR & Steroid profile		Predicted				Diagnostic performance†			
	Group	PHT	Bilateral PA	KCNJ5 WT	KCNJ5 MUT	Sensitivity		Specificity	
Actual	PHT	**142**	15	0	1	142/158	89.9%	222/273	81.3%
	Bilateral PA	39	**75**	16	4	75/134	56.0%	239/297	80.5%
	KCNJ5^{WT}	11	32	**32**	6	32/81	39.5%	322/350	92.0%
	KCNJ5^{MUT}	1	11	12	**34**	34/58	58.6%	362/373	97.1%

*The eight steroids in the selected profile include aldosterone, 18-oxocortisol, 18-hydroxycortisol, 11-deoxycorticosterone, cortisol, cortisone, androstenedione and DHEA. †Diagnostic sensitivities are specific to the indicated group, with specificities derived for all other groups; thus, the diagnostic sensitivities for identification of all patients with PA are indicated by specificities for the PHT group. Data are derived from analyses associated with the ROC curve analyses of Figure 3.Correct predictions for each group are indicated by data with grey backgrounds.

Batch effect removal assessment

In order to quantify the effects of batch corrections on the data, we implemented two different strategies of evaluation. In the first strategy we computed the AUC for each steroid, which provides a two-class separation measure between the two batches. This analysis was repeated for the raw dataset and the three different batch corrected datasets. The results of this analysis are summarized in eFigure 3, where the steroids (x-axis) are ordered for increasing AUC values (y-axis) of the raw dataset. All methods reduce in a general framework the batch effect, indeed before batch correction nine steroids were significantly impacted (we took as reference an AUC of 0.6). Those steroids largely also corresponded to the order of steroids indicated to show potential batch effects according to the least squares multivariate analyses outlined earlier. After correction for potential batch effects, four steroids remained impacted for RD, compared two for PPCCA and one for combat (eFugure 3 panels A, C and E). Note that the "batch influence" (measured as AUC value) after batch correction of single steroids may also simply reflect natural biological segregation of the four classes. Steroids are differently adjusted by the correction process, indeed the differences of aldosterone in the case of RD, progesterone in the case of Combat and 18-oxocortisol in the case of PPCCA, increase across the two batches. However, this differentiation could also have relation with the different biological classes, and not necessarily the different batches. Certainly, as outlined earlier the second batch

© 2020 Eisenhofer G et al. *JAMA Network Open.*

is that which only contains samples from patients with primary hypertension; nevertheless, it is stressed that these three employed batch correction methods are designed to address corrections with unbalanced classes between batches, such as in the present analysis.

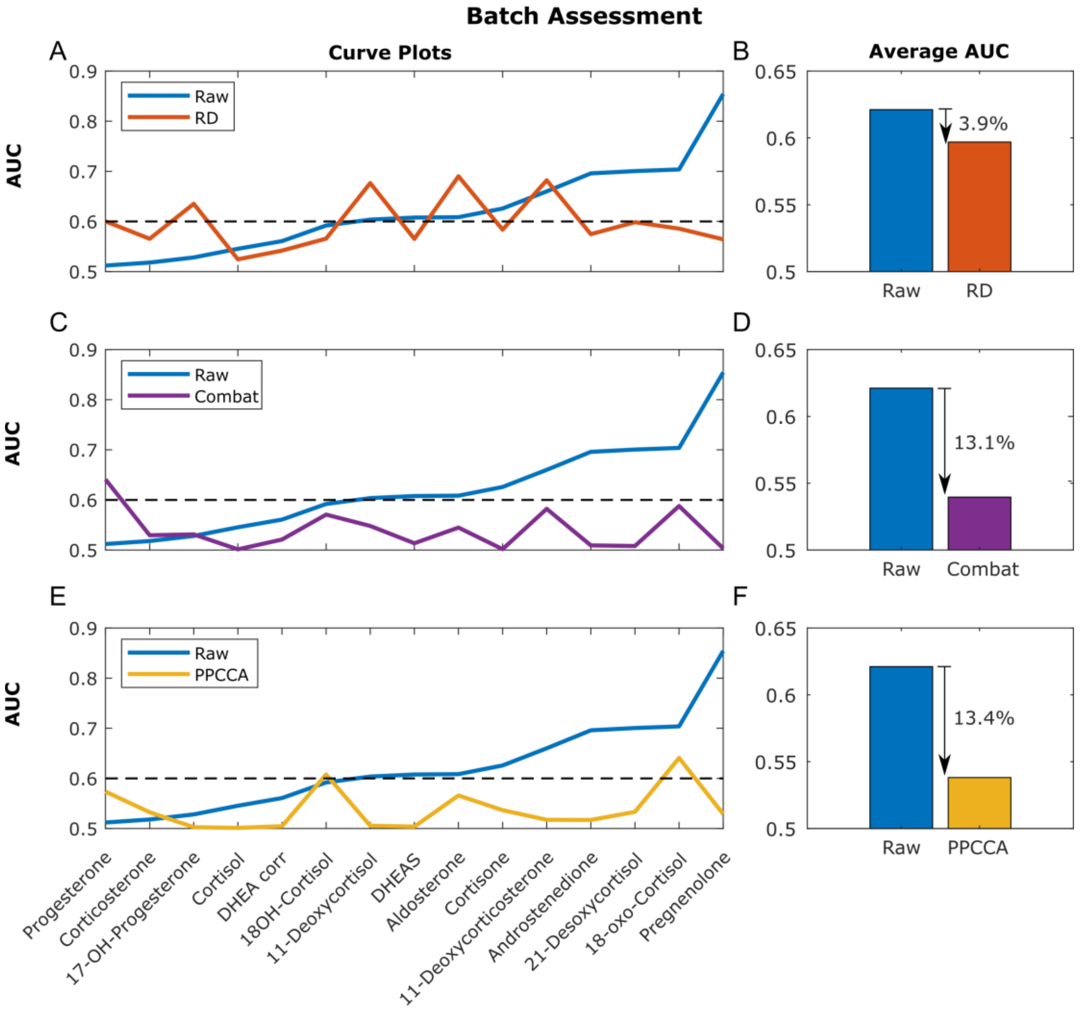

eFigure 3. AUC for the Assessment of Batch Influence and Correction for Each Steroid
The blue line in panels A, C and E corresponds to the AUC of the steroids without batch correction and is used to increasingly rank the steroids. The orange line in panel A corresponds to the AUC of the steroids with ratios of difference (RD) batch correction. The purple line in panel C corresponds to the AUC of the steroids with combat batch correction. The yellow line in panel E corresponds to the AUC of the steroids with PPCCA batch correction. The dashed lines with value 0.6 represent the threshold of AUC. The percentage decreases of the average AUC across the features for the data without batch correction (raw) and the batch correction methods are shown in panels B, D and F.

The overall quantification of the reduction of batch effect by the application of each correction method can be appreciated in eFigure 3 (panels B, D and F). Here, the average areas across the features (steroids) of the plots in panels A,C and E are calculated, and a decrease in percentages is appreciated for all batch correction methods, the most effective for PPCCA with a 13.4% of reduction, followed by Combat with 13.1% and RD with 3.9%.

In order to further evaluate batch effects, we also implemented a second strategy of evaluation: the Forest plots - outputs from the exploBATCH tool – from the data before and after correction are shown in eFigure 4. Here, the influence of the batch effect is measured for each of the four datasets (raw and three corrections) at each probabilistic principal component (pPCA). The closer the value of the point towards zero, the less

© 2020 Eisenhofer G et al. *JAMA Network Open.*

effect produced by the batches. The line represents the 95% confidence interval. Once again, PPCCA seems to be the most effective method for batch correction, followed by Combat and RD.

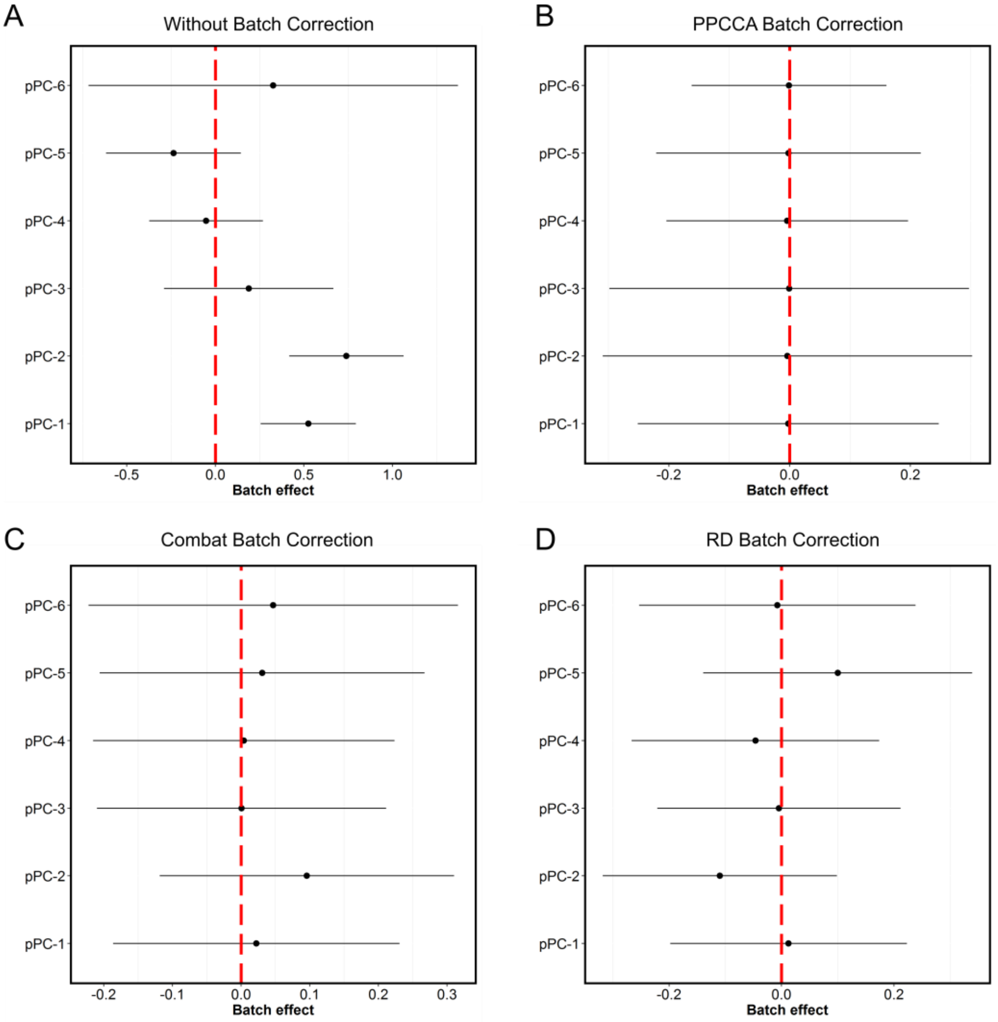

eFigure 4. Outputs Forest Plots by the Tool exploBATCH Quantifying the Batch Effects. A. Quantification of batch effect before correction. B. Quantification of batch effect after PPCCA correction. C. Quantification of batch effect after combat correction. D. Quantification of batch effect after RD correction

Machine learning results summary

Fifty-four models were selected from a total of 585 derived from nine different ML algorithms examined according to thirteen combinations of methods for data preparation (batch corrections and normalizations) and criteria for model evaluation, as well as the five learning proportions (LP) representing tested proportions for different training and test; and external validation sets (eFigures 5-10). Between five and seven features (steroids) were selected for each model. Models included some repetitions resulting from different criteria pointing to the same best model. Consequently after removal of model repetitions there was a final total of 21 unique top performing models (eTable 7). As a result of the use of the four feature selections (RFE, Gini, PLS and EN) employed in combination with similar normalization methods and batch corrections, selected features were identical for several of the final 21 unique top performing models.

© 2020 Eisenhofer G et al. *JAMA Network Open.*

Models 1-9: Criteria 1 (KCNJ5 mutation+ve unilateral disease and PA versus primary hypertension) performance by AUC

No	Model	Norm method	Batch Correction	TP	Selelcted features (steroids) in order of selection						
					1	2	3	4	5	6	7
1	SVMl-RFE	DRS	Combat	90/10	18OXOF	18OHF	CORT	F	E	DHEA	DHEAS
2	SVMl-EN	LOG-FM	RD	70/30	ALDO	18OXOF	18OHF	DOC	DHEA		
3	SVMnl-RFE	LOG-FM	RD	90/10	ALDO	18OXOF	18OHF	DOC	E	S	AE
4	SVMnl-EN	LOG-FM	RD	90/10	ALDO	18OXOF	18OHF	DOC	DHEA		
5	RF-Gini	DRS	PPCCA	90/10	ALDO	18OXOF	18OHF	CORT	DOC	17-OHP	DHEA
6	RF-EN	LOG-FM	RD	90/10	ALDO	18OXOF	18OHF	DOC	DHEA		
7	PLSDA	DCS	Combat	90/10	18OXOF	21DF	CORT	DOC	S	17-OHP	DHEA
8	LDA-EN	LOG-FM	RD	90/10	ALDO	18OXOF	18OHF	DOC	DHEA		
9	LR-EN	LOG-FM	RD	90/10	ALDO	18OXOF	18OHF	DOC	DHEA		

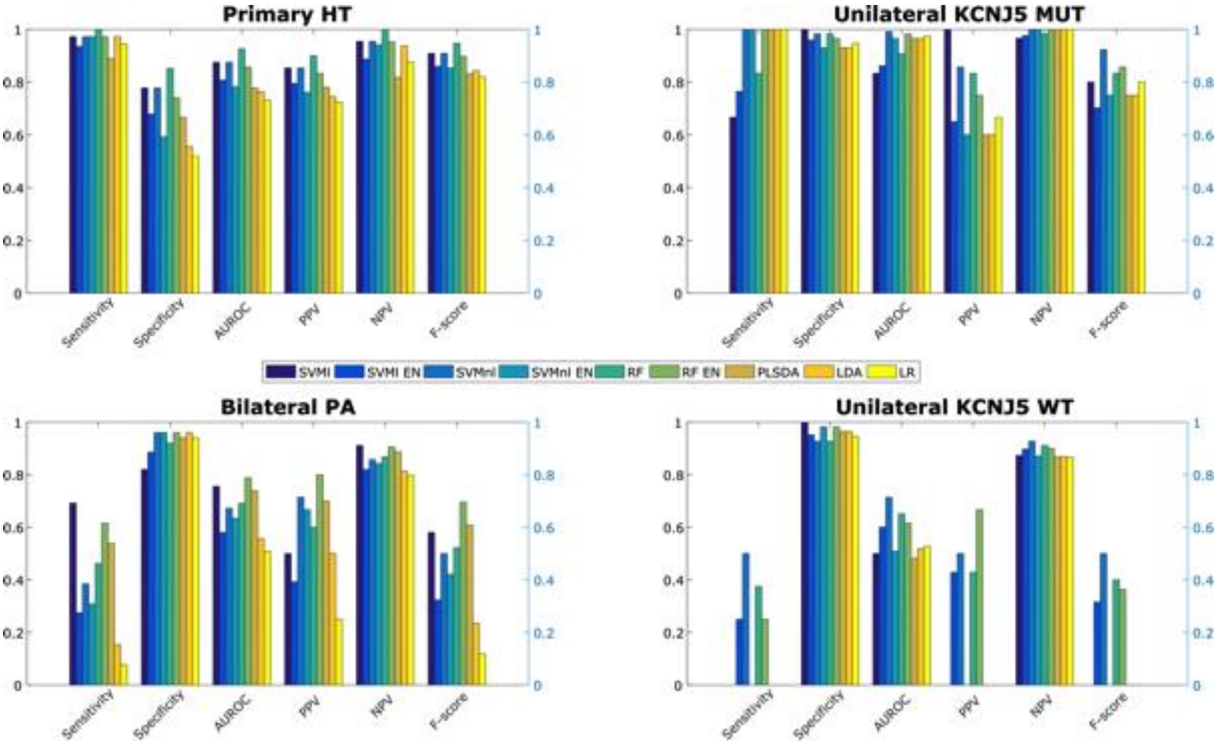

eFigure 5. Listings of Models With Selected Features and Bar Graphs of Diagnostic Performance (External Validation Series) for the First Set of Nine ML Algorithms According to Criteria 1 and Evaluations by AUC. Performance measures include diagnostic sensitivity and specificity, areas under ROC curves (AUROC) positive predictive values (PPV), negative predictive values (NPV) and F-scores. For abbreviations for models see eTable 5. 18OXOF, 18-oxocortisol; ALD, aldosterone; 18OHF, 18-hydroxycortisol; 21DF, 21-deoxycortisol; CORT, corticosterone; E, cortisone; DHEA, dehydroepiandrosterone; DHEAS, DHEA sulfate; DOC, 11-deoxycorticosterone; S, 11-deoxycortisol; 17-OHP, 17-hydroxyprogesterone; F, cortisol; AE, androstenedione.

Aldosterone, 18-oxocortisol and 18-hydroxycortisol in descending order occupied the top three selected places in nearly half of all unique models (eTable 7). This configuration was universally observed for RF-Gini and SVNnl-RFE models. The PLSDA model differed by placing 18-oxocortisol in top place, followed by 21-deoxycortisol and corticosterone. Aldosterone was also omitted from selected features in one of the two SVMl-RFE models, which featured 18-oxocortisol in top place, followed by 18-hydroxycortisol and corticosterone. The other SVMl-RFE model featured aldosterone, 18-oxocortisol and 21-deoxycortisol in the top three positions. Two of the LDA-EN models, two of the RF-EN models and one each of the SVMl-EN and SVMnl-EN models also included aldosterone, 18-oxocortisol and 18-hydroxycortisol in the top three selected places. Aldosterone was followed by 18-hydroxycortisol and 11-deoxycorticosterone in models that utilized EN for feature selection and LOG normalizations and PPCCA for batch correction. For models that employed EN for feature selection with SQRT and Combat for data prepration, 18-oxocortisol, 18-hydroxycortisol and 21-deoxycortisol occupied the top three positions.

© 2020 Eisenhofer G et al. *JAMA Network Open.*

Models 10-18: Criteria 2 (all three PA groups vs primary hypertension) performance evaluated by AUC

No	Model	Norm method	Batch Correction	TP	Selelcted features (steroids) in order of selection						
					1	2	3	4	5	6	7
10	SVMl-RFE	DRS	Combat	90/10	18OXOF	18OHF	CORT	F	E	DHEA	DHEAS
11	SVMl-EN	LOG-FM	RD	70/30	ALDO	18OXOF	18OHF	DOC	DHEA		
12	SVMnl-RFE	LOG-FM	RD	90/10	ALDO	18OXOF	18OHF	DOC	E	S	AE
13	SVMnl-EN	LOG	PPCCA	90/10	ALDO	18OHF	DOC	F	AE	DHEA	
14	RF-Gini	DRS	PPCCA	90/10	ALDO	18OXOF	18OHF	CORT	DOC	17-OHP	DHEA
15	RF-EN	LOG	PPCCA	90/10	ALDO	18OHF	DOC	F	AE	DHEA	
16	PLSDA	DCS	Combat	90/10	18OXOF	21DF	CORT	DOC	S	17-OHP	DHEA
17	LDA-EN	LOG-FM	RD	60/40	ALDO	18OXOF	18OHF	S	DHEA		
18	LR-EN	LOG-FM	RD	70/30	ALDO	18OXOF	18OHF	DOC	DHEA		

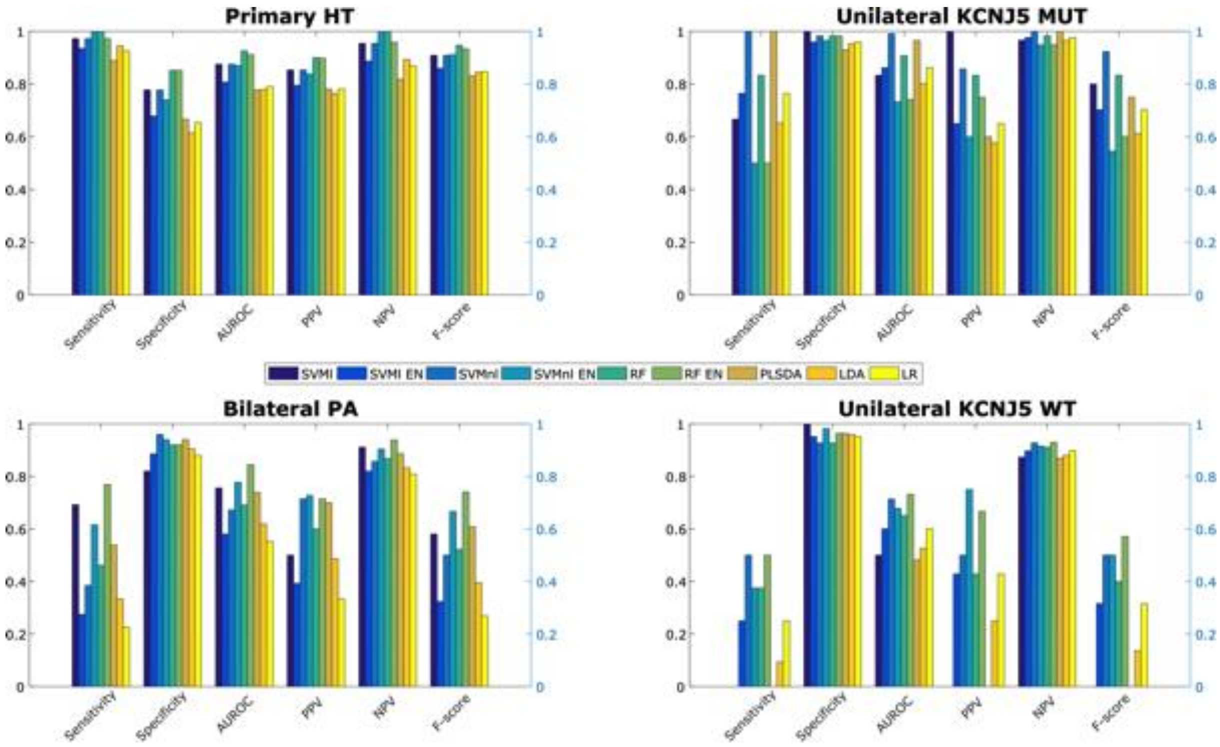

eFigure 6. Listings of Models With Selected Features and Bar Graphs of Diagnostic Performance (External Validation Series) for the Second Set of Nine ML Algorithms According to Criteria 2 and Evaluations by AUC. Performance measures include diagnostic sensitivity and specificity, areas under ROC curves (AUROC) positive predictive values (PPV), negative predictive values (NPV) and F-scores. For abbreviations for models see eTable 5. 18OXOF, 18-oxocortisol; ALD, aldosterone; 18OHF, 18-hydroxycortisol; 21DF, 21-deoxycortisol; CORT, corticosterone; E, cortisone; DHEA, dehydroepiandrosterone; DHEAS, DHEA sulfate; DOC, 11-deoxycorticosterone; S, 11-deoxycortisol; 17-OHP, 17-hydroxyprogesterone; F, cortisol; AE, androstenedione.

Corticosterone followed by 11-deoxycorticosterone, 17-hydroxyprogerone and DHEA occupied the next set of four selected features for the selected RF-Gini model (eFigures 5-10). In contrast, 11-deoxycorticosterone, cortisone, 11-deoxycortisol and androstenedione occupied the next set of selected features for the selected SVMnl-RFE model, whereas 11-deoxycorticosterone, 11-deoxycortisol, 17-hydroxyprogesterone and DHEA occupied those places in the PLSDA model. Interestingly, when aldosterone was not selected in five cases, four of them used Combat for batch correction, reflecting the importance of normalization and batch correction methods in the data preparation step. For LR-EN models, 11-deoxycorticosterone was followed by DHEA as selected features in fourth and fifth places with no further selections for all three variants of that model.

© 2020 Eisenhofer G et al. *JAMA Network Open.*

**Models 19-27:** Criteria 3 (best performance for KCNJ5 mutation+ve unilateral disease) performance evaluated by AUC											
No	Model	Norm method	Batch Correction	TP	Selelcted features (steroids) in order of selection						
					1	2	3	4	5	6	7
19	SVMl-RFE	Zscore	Combat	90/10	ALDO	18OXOF	21DF	CORT	DOC	S	17-OHP
20	SVMl-EN	SQRT	Combat	90/10	18OXOF	18OHF	21DF	DOC	F	DHEA	
21	SVMnl-RFE	LOG-FM	RD	90/10	ALDO	18OXOF	18OHF	DOC	E	S	AE
22	SVMnl-EN	LOG-FM	RD	90/10	ALDO	18OXOF	18OHF	DOC	DHEA		
23	RF-Gini	DRS	PPCCA	90/10	ALDO	18OXOF	18OHF	CORT	DOC	17-OHP	DHEA
24	RF-EN	LOG-FM	RD	90/10	ALDO	18OXOF	18OHF	DOC	DHEA		
25	PLSDA	DCS	Combat	90/10	18OXOF	21DF	CORT	DOC	S	17-OHP	DHEA
26	LDA-EN	LOG-FM	RD	90/10	ALDO	18OXOF	18OHF	DOC	DHEA		
27	LR-EN	LOG-FM	RD	90/10	ALDO	18OXOF	18OHF	DOC	DHEA		

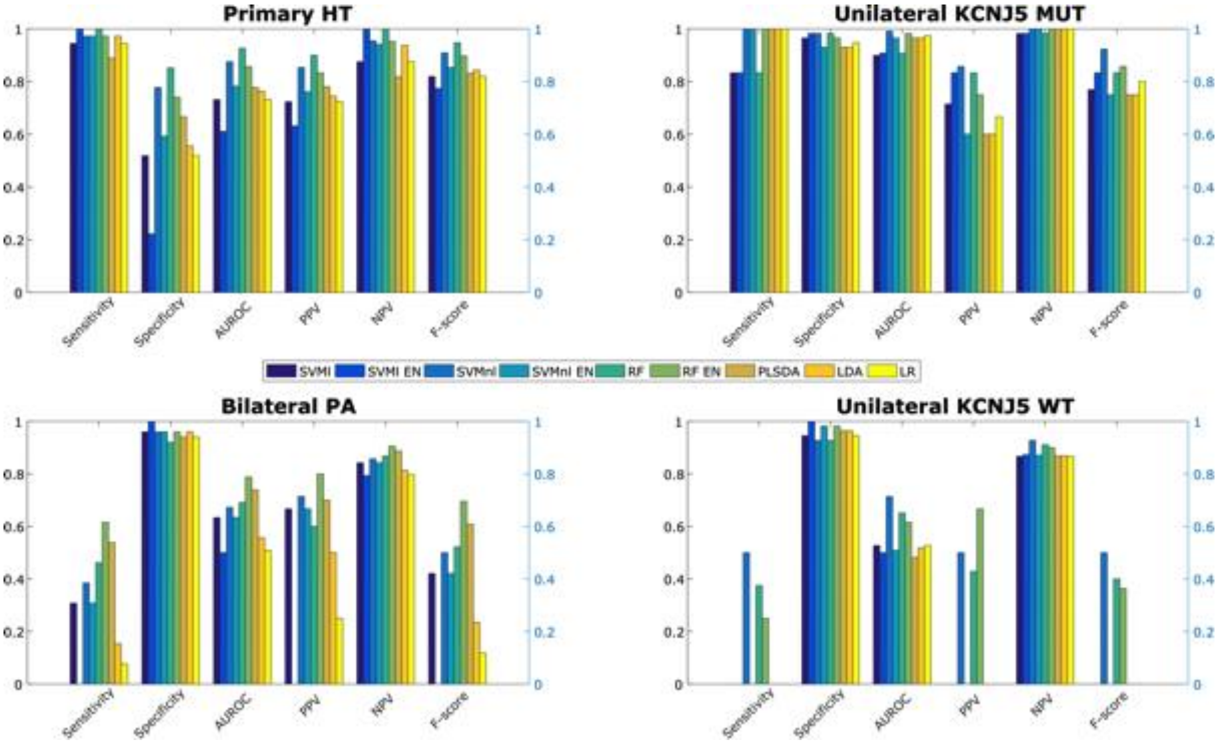

eFigure 7. Listings of Models With Selected Features and Bar Graphs of Diagnostic Performance (External Validation Series) for the Third Set of Nine ML Algorithms According to Criteria 3 and Evaluations by AUC. Performance measures include diagnostic sensitivity and specificity, areas under ROC curves (AUROC) positive predictive values (PPV), negative predictive values (NPV) and F-scores. For abbreviations for models see eTable 5. 18OXOF, 18-oxocortisol; ALD, aldosterone; 18OHF, 18-hydroxycortisol, 21DF, 21-deoxycortisol; CORT, corticosterone; E, cortisone; DHEA, dehydroepiandrosterone; DHEAS, DHEA sulfate; DOC, 11-deoxycorticosterone; S, 11-deoxycortisol; 17-OHP, 17-hydroxyprogesterone; F, cortisol; AE, androstenedione.

All other models varied in configurations of selected features for positions four through to seven and none included aldosterone, 18-oxocortisol or 18-hydroxycortisol at those positions (eFigures 5-10). The selected features occupying positions four or five in descending order of prevalence included 11-deoxycorticosterone, DHEA, cortisol, corticosterone, cortisone, 11-deoxycortisol, androstenedione and 21-deoxycortisol. Top steroids in places six and seven in descending order of prevalence were DHEA, 17-hydroxyprogesterone (, androstenedione, 11-deoxycortisol, DHEAS and 11-deoxycorticosterone.

© 2020 Eisenhofer G et al. *JAMA Network Open.*

No	Model	Norm method	Batch Correction	TP	Selelcted features (steroids) in order of selection						
					1	2	3	4	5	6	7
28	SVMl-RFE	DRS	Combat	90/10	18OXOF	18OHF	CORT	F	E	DHEA	DHEAS
29	SVMl-EN	SQRT	Combat	90/10	18OXOF	18OHF	21DF	DOC	F	DHEA	
30	SVMnl-RFE	LOG-FM	RD	90/10	ALDO	18OXOF	18OHF	DOC	E	S	AE
31	SVMnl-EN	DCS	PPCCA	90/10	ALDO	18OXOF	21DF	DOC	F	AE	DHEA
32	RF-Gini	DRS	PPCCA	90/10	ALDO	18OXOF	18OHF	CORT	DOC	17-OHP	DHEA
33	RF-EN	SQRT	PPCCA	90/10	ALDO	18OXOF	18OHF	21DF	CORT	DOC	DHEA
34	PLSDA	DRS	Combat	50/50	18OXOF	21DF	CORT	DOC	S	17-OHP	DHEA
35	LDA-EN	LOG-FM	RD	90/10	ALDO	18OXOF	18OHF	DOC	DHEA		
36	LR-EN	LOG-FM	RD	80/20	18OXOF	18OHF	21DF	DOC	DHEA		

Table title: **Models 28-36**: Criteria 1 (KCNJ5 mutation+ve unilateral disease and PA versus primary hypertension) performance by F-score

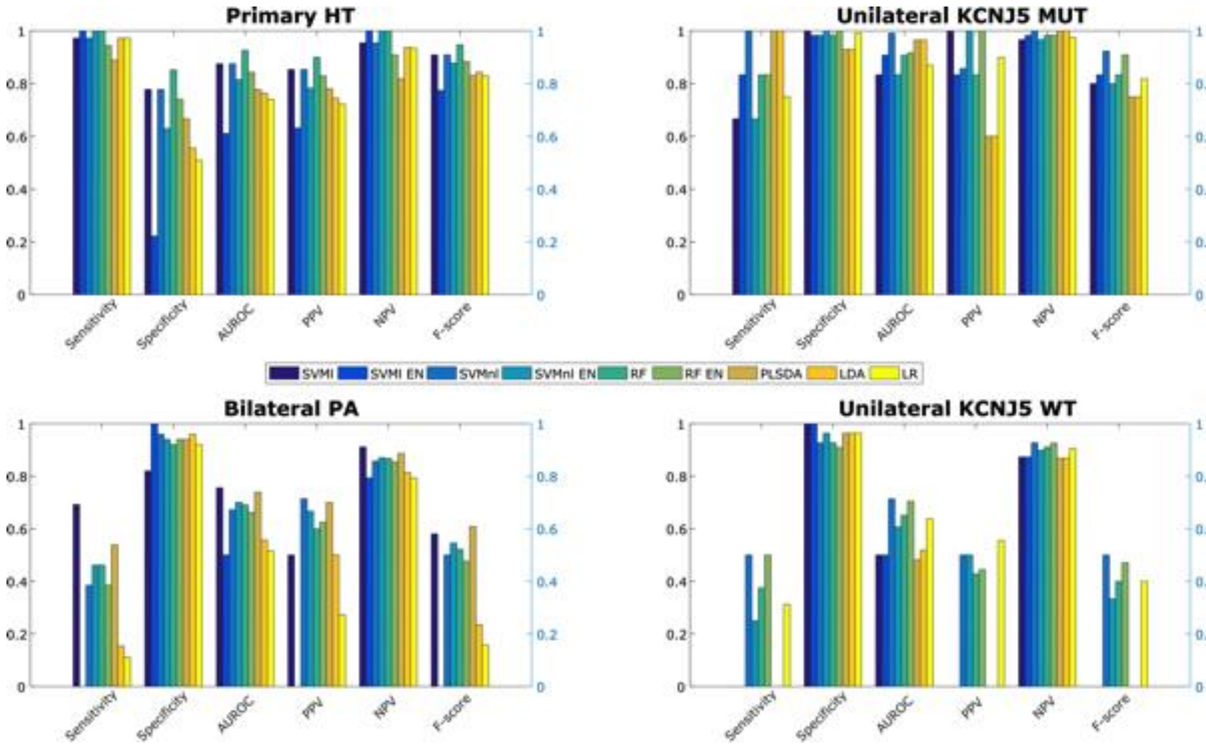

eFigure 8. Listings of Models With Selected Features and Bar Graphs of Diagnostic Performance (External Validation Series) for the Fourth Set of Nine ML Algorithms According to Criteria 3 and Evaluations by F-Score. Performance measures include diagnostic sensitivity and specificity, areas under ROC curves (AUROC) positive predictive values (PPV), negative predictive values (NPV) and F-scores. For abbreviations for models see eTable 5. 18OXOF, 18-oxocortisol; ALD, aldosterone; 18OHF, 18-hydroxycortisol, 21DF, 21-deoxycortisol; CORT, corticosterone; E, cortisone; DHEA, dehydroepiandrosterone; DHEAS, DHEA sulfate; DOC, 11-deoxycorticosterone; S, 11-deoxycortisol; 17-OHP, 17-hydroxyprogesterone; F, cortisol; AE, androstenedione.

Performance of ML models – assessed by diagnostic sensitivity and specificity, areas under ROC curves (AUROC), positive predictive values (PPV), negative predictive values (NPV) and F-scores – varied considerably according to class (i.e., primary hypertension, bilateral PA and $KCNJ5^{MUT}$ and $KCNJ5^{WT}$), but less so among the nine ML algorithms or according to the different normalizations, batch corrections and criteria for performance evaluations (eFigures 5-10). Independent of those latter criteria and considering only the 21 unique models (eTable 7), diagnostic performance, as assessed by AUROC, was higher (0.885±0.034 vs 0.793±0.039, P<0.05) for identifying patients with *KCNJ5* mutated APAs compared to identifying those with primary hypertension (i.e., distinguishing patients with PA from those with primary hypertension). This reflected higher diagnostic sensitivity (79.8±6.8% vs 62.0±7.8%, P<0.05) for identification of patients with *KCNJ5* mutated APAs compared to identification of those with PA at equivalent levels of specificity (97.2±1.0% vs 96.7±1.3%). Diagnostic performance as assessed by AUROC

© 2020 Eisenhofer G et al. *JAMA Network Open.*

was considerably lower (P<0.005) than the above measures for identification of respective patients with *KCNJ5* wildtype APAs and bilateral PA (0.597±0.035 and 0.651±0.050).

Models 37-45: Criteria 2 (all three PA groups vs primary hypertension) performance evaluated by F-score

No	Model	Norm method	Batch Correction	TP	Selelcted features (steroids) in order of selection						
					1	2	3	4	5	6	7
37	SVMl-RFE	DRS	Combat	90/10	18OXOF	18OHF	CORT	F	E	DHEA	DHEAS
38	SVMl-EN	LOG-FM	RD	70/30	ALDO	18OXOF	18OHF	DOC	DHEA		
39	SVMnl-RFE	LOG-FM	RD	90/10	ALDO	18OXOF	18OHF	DOC	E	S	AE
40	SVMnl-EN	LOG	PPCCA	90/10	ALDO	18OHF	DOC	F	AE	DHEA	
41	RF-Gini	DRS	PPCCA	90/10	ALDO	18OXOF	18OHF	CORT	DOC	17-OHP	DHEA
42	RF-EN	No-norm	PPCCA	90/10	ALDO	18OXOF	21DF	DOC	F	AE	DHEA
43	PLSDA	DRS	Combat	90/10	18OXOF	21DF	CORT	DOC	S	17-OHP	DHEA
44	LDA-EN	LOG-FM	RD	60/40	ALDO	18OXOF	18OHF	S	DHEA		
45	LR-EN	LOG-FM	RD	70/30	ALDO	18OXOF	18OHF	DOC	DHEA		

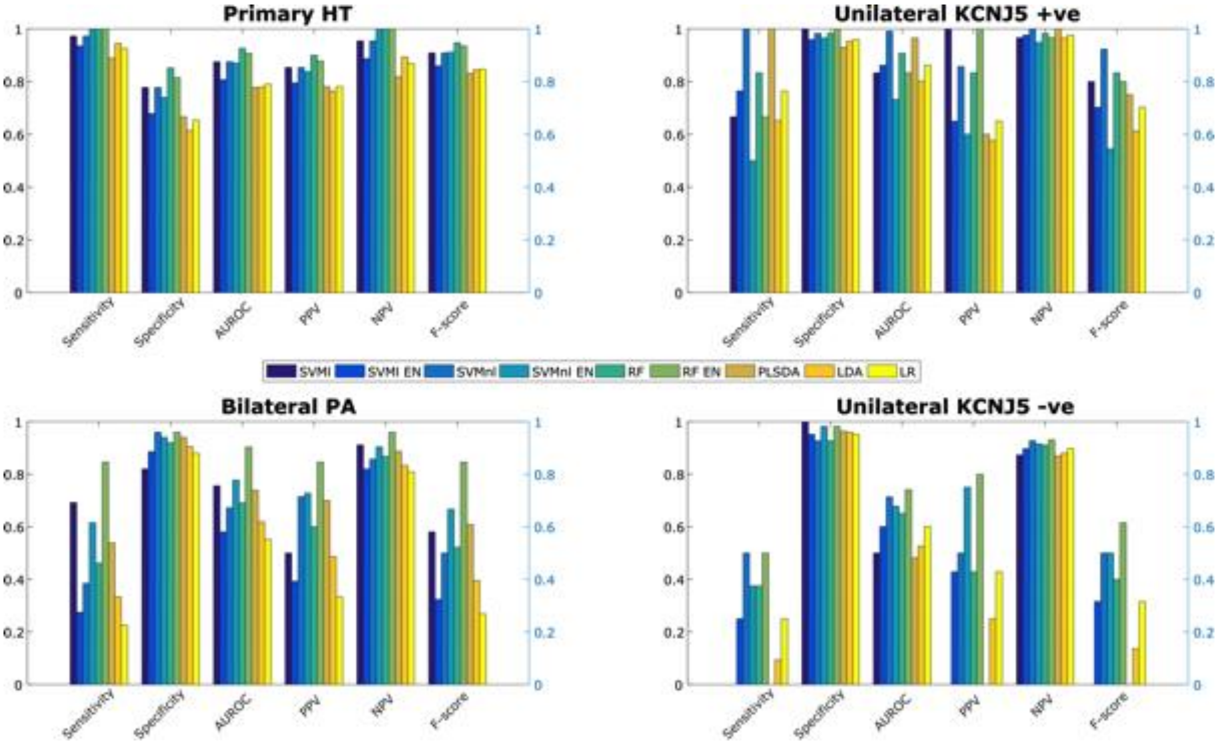

eFigure 9. Listings of Models With Selected Features and Bar Graphs of Diagnostic Performance (External Validation Series) for the Fifth Set of Nine ML Algorithms According to Criteria 2 and Evaluations by F-Score. Performance measures include diagnostic sensitivity and specificity, areas under ROC curves (AUROC) positive predictive values (PPV), negative predictive values (NPV) and F-scores. For abbreviations for models see eTable 5. 18OXOF, 18-oxocortisol; ALD, aldosterone; 18OHF, 18-hydroxycortisol, 21DF, 21-deoxycortisol; CORT, corticosterone; E, cortisone; DHEA, dehydroepiandrosterone; DHEAS, DHEA sulfate; DOC, 11-deoxycorticosterone; S, 11-deoxycortisol; 17-OHP, 17-hydroxyprogesterone; F, cortisol; AE, androstenedione.

Although most of the selected top performing 21 unique models (eTable 7) performed comparably well for identification of patients with PA or *KCNJ5* mutated APAs, some models performed a little better than others and there were other differences indicating preference for some models over others for identifying specific patient classes. Among the 21 unique models the SVMnl-RFE, RF-Gini and four RF-EN models provided the best overall diagnostic performance for all classes in terms of AUROCs from 0.794 to 0.813 and F-scores from 0.676-0.725. In contrast LDA-EN, LR-EN and SVMl-EN models were the those that performed more poorly with AUROCs across all classes from 0.671 to 0.693 and F-scores from 0.477 to 0.506.

© 2020 Eisenhofer G et al. *JAMA Network Open.*

Models 46-54: Criteria 3 (best performance for KCNJ5 mutation+ve unilateral disease) performance evaluated by F-score

No	Model	Norm method	Batch Correction	TP	Selelcted features (steroids) in order of selection						
					1	2	3	4	5	6	7
46	SVMl-RFE	DRS	Combat	90/10	18OXOF	18OHF	CORT	F	E	DHEA	DHEAS
47	SVMl-EN	SQRT	Combat	90/10	18OXOF	18OHF	21DF	DOC	F	DHEA	
48	SVMnl-RFE	LOG-FM	RD	90/10	ALDO	18OXOF	18OHF	DOC	E	S	AE
49	SVMnl-EN	SQRT	Combat	90/10	18OXOF	18OHF	21DF	DOC	F	DHEA	
50	RF-Gini	DRS	PPCCA	90/10	ALDO	18OXOF	18OHF	CORT	DOC	17-OHP	DHEA
51	RF-EN	SQRT	PPCCA	90/10	ALDO	18OXOF	18OHF	21DF	CORT	DOC	DHEA
52	PLSDA	DRS	Combat	90/10	18OXOF	21DF	CORT	DOC	S	17-OHP	DHEA
53	LDA-EN	LOG	PPCCA	90/10	ALDO	18OHF	DOC	F	AE	DHEA	
54	LR-EN	LOG-FM	RD	80/20	18OXOF	18OHF	21DF	DOC	DHEA		

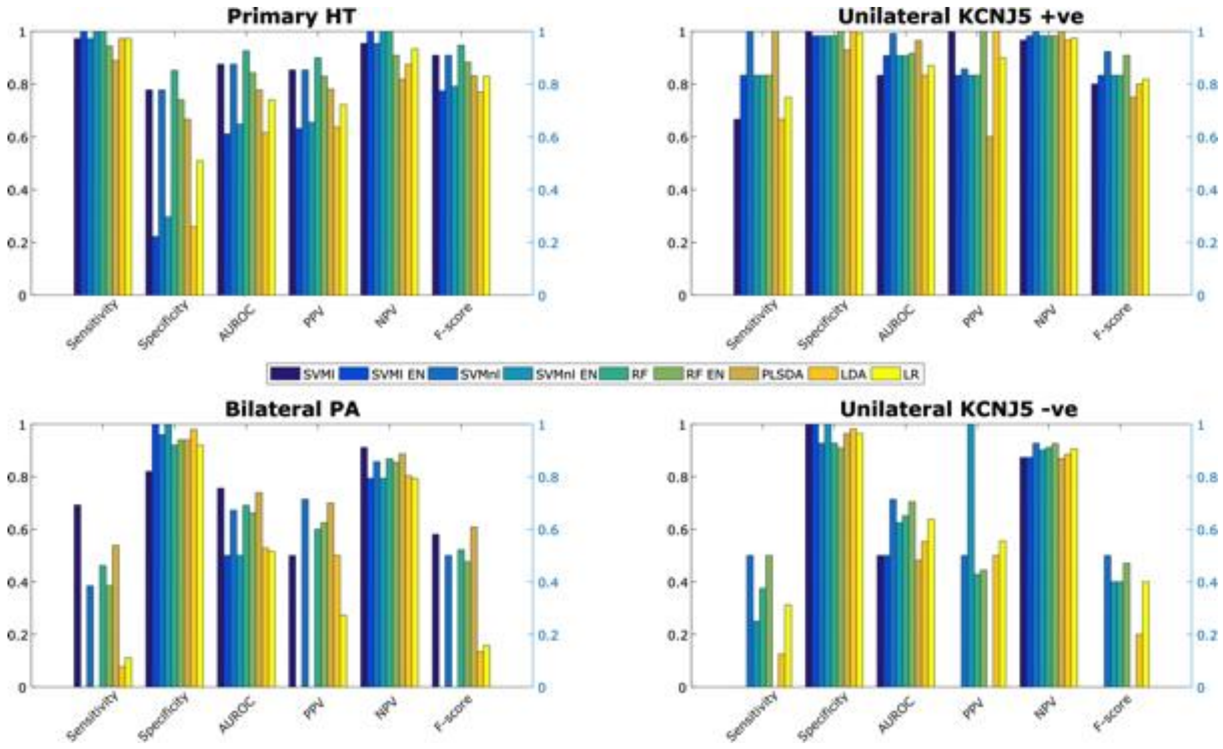

eFigure 10. Listings of Models With Selected Features and Bar Graphs of Diagnostic Performance (External Validation Series) for the Sixth Set of Nine ML Algorithms According to Criteria 2 and Evaluations by F-Score. Performance measures include diagnostic sensitivity and specificity, areas under ROC curves (AUROC) positive predictive values (PPV), negative predictive values (NPV) and F-scores. For abbreviations for models see eTable 5. 18OXOF, 18-oxocortisol; ALD, aldosterone; 18OHF, 18-hydroxycortisol, 21DF, 21-deoxycortisol; CORT, corticosterone; E, cortisone; DHEA, dehydroepiandrosterone; DHEAS, DHEA sulfate; DOC, 11-deoxycorticosterone; S, 11-deoxycortisol; 17-OHP, 17-hydroxyprogesterone; F, cortisol; AE, androstenedione

Among the top performers, RF-EN models performed well for identification of all classes, but the RF-Gini model provided highest performance for diagnosis of PA with an AUROC of 0.926, and F-value of 0.947 and diagnostics sensitivities and specificities of 85% and 100%. The SVMnl-RFE model provided best performance for identification of patients with *KCNJ5* mutation+ve APAs according to an AUROC of 0.991, and F-ratio of 0.923 and diagnostics sensitivities and specificities of 100% and 98%.

© 2020 Eisenhofer G et al. *JAMA Network Open.*

**eTable 7.** Final 21 Unique Top Performing ML Models											
Model	No Rep	Norm method	Batch correction	LP	Selected features (steroids) in order of selection						
					1	2	3	4	5	6	7
SVMl-RFE	5	DRS	Combat	90/10	18OXOF	18OHF	CORT	F	E	DHEA	DHEAS
SVMl-RFE	1	Zscore	Combat	90/10	ALDO	18OXOF	21DF	CORT	DOC	S	17-OHP
SVMl-EN	3	LOG-FM	RD	70/30	ALDO	18OXOF	18OHF	DOC	DHEA		
SVMl-EN	3	SQRT	Combat	90/10	18OXOF	18OHF	21DF	DOC	F	DHEA	
SVMnl-RFE	6	LOG-FM	RD	90/10	ALDO	18OXOF	18OHF	DOC	E	S	AE
SVMnl-EN	2	LOG-FM	RD	90/10	ALDO	18OXOF	18OHF	DOC	DHEA		
SVMnl-EN	2	LOG	PPCCA	90/10	ALDO	18OHF	DOC	F	AE	DHEA	
SVMnl-EN	1	DCS	PPCCA	90/10	ALDO	18OXOF	21DF	DOC	F	AE	DHEA
SVMnl-EN	1	SQRT	Combat	90/10	18OXOF	18OHF	21DF	DOC	F	DHEA	
RF-Gini	6	DRS	PPCCA	90/10	ALDO	18OXOF	18OHF	CORT	DOC	17-OHP	DHEA
RF-EN	2	LOG-FM	RD	90/10	ALDO	18OXOF	18OHF	DOC	DHEA		
RF-EN	1	LOG	PPCCA	90/10	ALDO	18OHF	DOC	F	AE	DHEA	
RF-EN	2	SQRT	PPCCA	90/10	ALDO	18OXOF	18OHF	21DF	CORT	DOC	DHEA
RF-EN	1	NoNorm	PPCCA	90/10	ALDO	18OXOF	21DF	DOC	F	AE	DHEA
PLSDA	6	DCS	Combat	90/10	18OXOF	21DF	CORT	DOC	S	17-OHP	DHEA
LDA-EN	3	LOG-FM	RD	90/10	ALDO	18OXOF	18OHF	DOC	DHEA		
LDA-EN	2	LOG-FM	RD	60/40	ALDO	18OXOF	18OHF	S	DHEA		
LDA-EN	1	LOG	PPCCA	90/10	ALDO	18OHF	DOC	F	AE	DHEA	
LR-EN	2	LOG-FM	RD	90/10	ALDO	18OXOF	18OHF	DOC	DHEA		
LR-EN	2	LOG-FM	RD	70/30	ALDO	18OXOF	18OHF	DOC	DHEA		
LR-EN	2	LOG-FM	RD	80/20	18OXOF	18OHF	21DF	DOC	DHEA		

Abbreviations: No Rep, numbers of replicates; Norm Method, normalization method; LP, learning proportion; SVM, support vector machine; RF, random forest; PLSDA, partial least square discriminant analysis; LDA, linear discriminant analysis; LR, logistic regression; l, linear; nl, non-linear; RFE, recursive feature elimination; EN, elastic net; DRS, division of each cell-row by its row sum; LOG-FM, logarithm to the base10 of the fold-difference of concentrations from an age and sex-specific reference population mean; DCS, division of each cell-column by its column sum; RD, ratios of differences; PPCCA , probabilistic principal component and covariate analysis; No Norm, no normalization. 18OXOF, 18-oxocortisol; ALD, aldosterone; 18OHF, 18-hydroxycortisol; 21DF, 21-deoxycortisol; CORT, corticosterone; E, cortisone; DHEA, dehydroepiandrosterone; DHEAS, DHEA sulfate; DOC, 11-deoxycorticosterone; S, 11-deoxycortisol; 17-OHP, 17-hydroxyprogesterone; F, cortisol; AE, androstenedione.

The top two models (RF-Gini and SVMnl-RFE) according to both overall performance for classification of all classes and then specifically best performance for distinguishing PA from primary hypertension and identifying unilateral *KCNJ5* mutated APAs were selected at learning to external validation proportions of 90% to 10%. Thus, at those proportions external validations were performed in 63 patients and values for diagnostic performance represent best estimate probabilities relative to the learning series. At lower learning proportions, values for the various measures diagnostic performance in external validation series showed overall alignment below or above values for performance measures in learning series for the SVMnl-RFE model (eFigure 11) and the RF-Gini model (eFigure 12). However, the RF-Gini model showed overall closer alignment and less variation between learning and external validation measures of performance compared to the SVMnl-RFE model. This reflected the similar numbers of patients with PA compared to patients with primary hypertension for evaluating performance in distinguishing those two groups. In contrast, patients with *KCNJ5* mutated APAs represented slightly less than 10% of all patients; thus, learning and validation series for identification of these patients were not only less closely aligned than for the RF-Gini identification of patients with PA, but also showed a trend towards increasing values for diagnostic performance at higher than lower learning ratios.

© 2020 Eisenhofer G et al. *JAMA Network Open.*

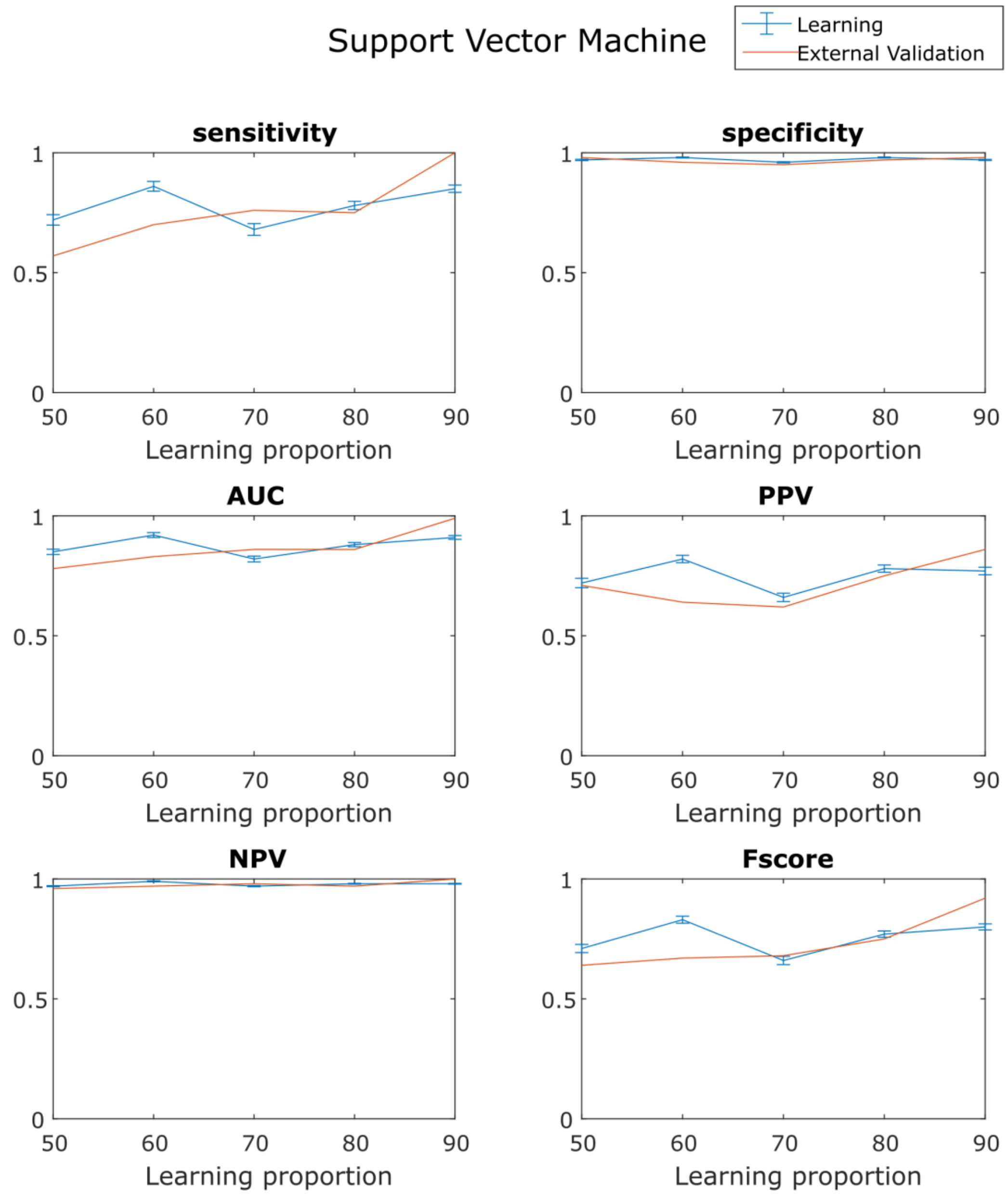

eFigure 11. Measures of Diagnostic Performance for Identification of Patients With *KCNJ5* Mutation+ve APAs Using the SVMnl-RFE Model. Measures are shown for learning series (with confidence intervals) and external validation series at five different learning proportions of 50/50, 60/40, 70/30, 80/20 and 90/10.

© 2020 Eisenhofer G et al. *JAMA Network Open.*

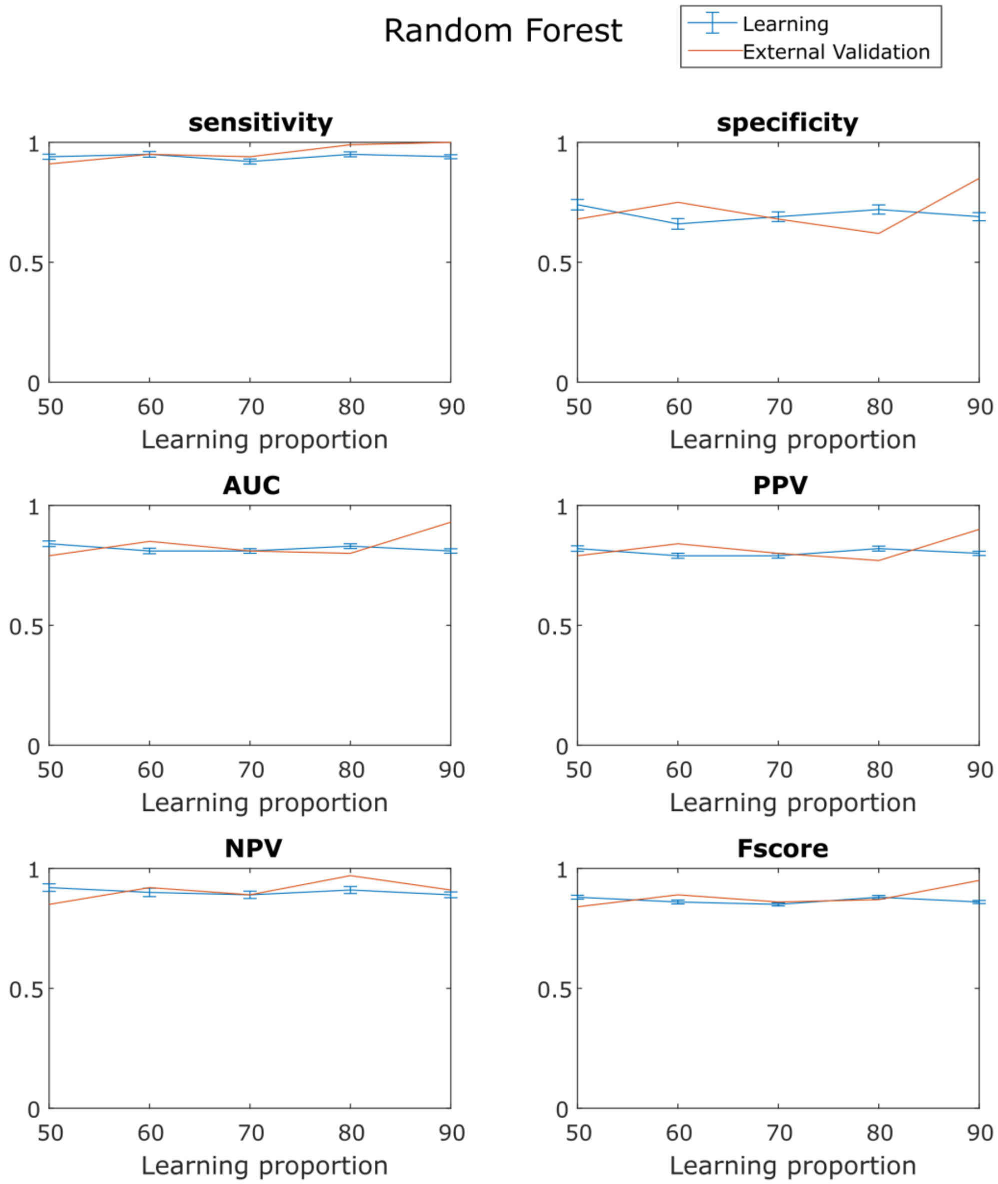

eFigure 12. Measures of Diagnostic Performance for Identification of Patients With Primary Hypertension Using the RF-Gini Model. Measures are shown for learning series (with confidence intervals) and external validation series at five different learning proportions of 50/50, 60/40, 70/30, 80/20 and 90/10. Note that measures of diagnostic performance are shown for identification of patients with primary hypertension, so that for identification of patients with PA (compared to primary hypertension) diagnostic sensitivities and specificities as well as PPVs and NPVs must be reversed.

© 2020 Eisenhofer G et al. *JAMA Network Open.*

eAppendix 3. Supplemental Discussion

The present study extends our groups previous work on steroid profiling in patients with PA[1,2,13] by establishing that the combination of LC-MS/MS based measurements of steroid profiles with ML can potentially be used at screening to both improve identification of patients with PA and in the same screening step identify those patients most likely to benefit from subsequent diagnostic and therapeutic interventions. Diagnostic interventions include AVS, a technically demanding expensive and invasive radiological procedure used according to current guidelines to discriminate bilateral from unilateral PA and thereby identify the latter patients for adrenalectomy. However, as we show here and also in agreement with other recent reports, a significant proportion of patients identified by AVS for adrenalectomy are not cured by this intervention. Also in agreement with previous findings of other groups, patients with aldosterone-producing adenomas associated with somatic *KCNJ5* mutations show the most benefit from adrenalectomy. As we now establish here for the first time, those patients with aldosterone-producing adenomas due to *KCNJ5* mutations can be easily identified among patients with primary hypertension and other patients with PA by highly distinctive steroids profiles at a diagnostic sensitivity of at least 85% with a specificity of at least 97%. The high positive predictive value of the combination of steroid profiling with ML enables patients with APAs due to *KCNJ5* mutations to be efficiently triaged for AVS or potentially even for immediate adrenalectomy in cases where imaging evidence indicates an adenoma localized to one adrenal.

Study limitations

Among study limitations related to the retrospective design, differences in seated versus supine sampling in different patient groups deserve special consideration. The higher plasma concentrations of aldosterone and 18-oxocortisol in patients with primary hypertension who were screened for PA compared to the patients with hypertension in the reference group likely reflect the known impacts of posture on plasma renin and aldosterone and sampling for steroid profiling in the seated position for most of the former group compared to the supine position for the latter group. Although this would not be expected to impact the diagnostic performance of the ARR (since sampling for those measurements was carried out seated in both primary hypertensive and PA populations), this may have adversely impacted the performance of steroid profiles for distinguishing groups with PA from primary hypertensives. This would be expected to occur since seated sampling of blood for steroid profiling of more than half of the patients with primary hypertension compared to supine sampling in 93% of patients with PA would be expected to reduce the difference in plasma aldosterone and possibly other steroids between groups with and without PA. Thus, the diagnostic power of steroid profiles both alone and compared to the ARR may have been underestimated for those comparisons. Improved diagnostic power of steroid profiles, both alone and compared to the ARR, could be expected if seated sampling had been employed for all analyses of plasma steroid profiles.

A second study limitation involved changes to LC-MS/MS assay methodology that resulted in differences before and after method optimization most apparent for measurements of plasma pregnenolone. For that reason pregnenolone was excluded from consideration in subsequent logistic regression and ML analyses. Furthermore, since samples from patients with primary hypertension were all analyzed after method optimization it was important to consider and correct potential batch differences before and after optimization. As summarized in eTable 4 potential batch differences were identified in numerous other steroids besides pregnenolone. For this reason batch corrections, as outlined in eTable 5, were employed to correct for batch effects. As described in the analyses associated with in eFigures 3 and 4, not all methods for batch correction were equally effective for all steroids. This may have contributed to inaccuracies in comparisons and algorithms particularly for patients with PA versus primary hypertension. Nevertheless, differences in most measured steroids according to group were much larger than those according to batch and any imprecisely corrected batch effect is therefore likely of minor relevance.

A third limitation relates to the need for an increased population size of the hypertensive control group to develop ML algorithms, which was achieved using additional patients from a hypertensive reference group in who measurements of the ARR were not available. This and the fact that many samples for the ARR were taken at different time points than those for steroid profiles therefore precluded considerations of the ARR or plasma renin in ML algorithms.

A further limitation involves the coverage of testing for *KCNJ5* mutations. Genotype data in this study, as also recognized in other studies, should be interpreted with caution because recent evidence shows that when sequencing is targeted to areas of the adenoma that are positive for CYP11B2 expression, somatic mutations are detected in almost 90% of APAs.[14] Therefore, the non-targeted sequencing approach used here is a limitation of the current study with some degree of overrepresentation of the *KCNJ5* “wild-type” genotype. This would have been expected again to adversely impact the power of steroid profiling to discriminate the groups of patients with unilateral disease with and without *KCNJ5* mutations and may have contributed to some overlap in steroid profiles in the two groups of patients.

Similarly, since initial classification of patients into groups for subsequent analyses — including ML, depended on imperfect routine diagnostic tests (i.e., ARR, saline suppression tests, AVS) it is possible that some patients with primary hypertension or bilateral PA may have been inappropriately classified. It is also possible that some patients in the reference hypertensive group may have had undiagnosed PA. Such inaccuracies would be expected again to lead to underestimates of diagnostic performance of steroid profiles for both diagnosis and subtype classification of PA.

Finally, without larger patient populations for further validations the true diagnostic performance of machine learning models is also not yet accurately determined, particularly for the SVM model where $KCNJ5^{MUT}$ cases were limited.

Future perspectives and challenges

Many of the above limitations can be addressed in new prospective studies involving patients screened for PA using both routine diagnostic tests (i.e, ARR) and LC-MS/MS-based steroid profiling. For such studies, the ML learning algorithms developed in the present study are now available for further validation and improvement. One such study (prospective study on the diagnostic value of steroid profiling in primary aldosteronism – PROSALDO – trial registration no DRKS00017084) underway at multiple centers in Europe and Australia involves comparisons of both steroid profiling and the ARR at screening to guide clinical decision-making. The study involves a laboratory information management system prototype developed for rapid and automated application of machine learning algorithms. Automated reports are generated that feature probabilities of PA versus PHT and when the former is indicated further probabilities of bilateral versus unilateral disease, including for the latter presence of KCNJ5 mutations. Raw data for steroid profiles, including sex and age specific reference intervals are also included in patient reports, which after generation are immediately made available to investigators at study centers. Based on either positive results for the steroid profile or the ARR, patients then undergo confirmatory saline infusion tests and when positive, AVS and/or surgical intervention the latter depending on steroid profiles, AVS results and/or imaging studies. Follow-up of operated patients and mutation testing is carried out to assess outcomes and respectively confirm or exclude unilateral disease and presence of KCNJ5 mutations. A first publication arising from this study illustrates some specific advantages of steroid profiling over the traditional multiple step process for diagnostic subtyping of PA.[15]

As outlined in the present report, steroid profilies may provide a solution to immediate identification of patients with APAs due to KCNJ5 mutations who show the most clinical benefit from surgical removal of the adrenal source of aldosterone hypersecretion. In such patients with a clearly defined unilateral adenoma found on imaging it may also be possible to proceed directly to surgical intervention without need for confirmatory saline infusion tests or AVS studies. However, at least in Caucasians, KCNJ5 mutations are responsible for only a minority of APAs. A meta-analysis indicated a 35% prevalence of KCNJ5-mutated APAs among Western populations compared to 63% prevalence among Chinese and Japanese populations[16] Another report, involving a sensitive strategy for testing KCNJ5 mutations suggested a higher prevalence of 43% among Caucasian Americans compared to a 34% prevalence KCNJ5-mutated APAs among African Americans.[17] For the present series the prevalence of KCNJ5 mutated APAs was 38%. As also shown, the presently employed steroid profiles do not provide an accurate method for distinguishing other patients with APAs from those with bilateral aldosterone producing disease and also fall short in accurately distinguishing patients with primary hypertension from those with PA. Nevertheless, the possibility to streamline the current labor-intensive multistep diagnostic process at least in 40% or more of patients with unilateral APAs would represent a good first step to more effective diagnosis and therapeutic intervention of a disease that remains poorly recognized duie to the difficulties in the diagnostic process.

© 2020 Eisenhofer G et al. *JAMA Network Open.*

As outlined by Funder,[18] less than one percent of patients with PA are ever identified, an observation that has received more recent support by Brown et al.,[19] who clarified that the prevalence of PA remains largely unrecognized. In the latter report there was additional emphasis on the poor diagnostic accuracy of the ARR, which is in agreement with observations of others[20] and findings in the present study of a diagnostic sensitivity of 84.5% at a specificity of only 71.5%. Combined with difficulties in dealing with the many variables that impact the ARR, including antihypertensive medications,[21] this indicates an unmet need for vastly improved strategies for screening for PA among patients with hypertension. Although the combination of ML with steroid profiling alone is unlikely to completely address this need, it is likely that diagnostic algorithms for both single step screening and subtyping may be further improved by incorporation of other measurements. As shown in the present report, renin represents the most obvious additional measurement, but it is also possible that other improvements may be obtained by inclusion of mass spectrometric measurements of angiotensin peptides,[22,23] which are purported to be less susceptible to medication associated problems than renin.[24]

As reviewed elsewhere,[25] challenges for wider clinical implementation of steroid profiling and ML include needs for harmonization of mass spectrometry-based steroid measurements and the requirements for considerable amounts of data from well characterized patient cohorts to further develop and improve ML algorithms. Integration of multidimensional diagnostic approaches with clinical and laboratory management information systems is another challenge that must be met before steroid profiling and ML can be introduced into routine diagnostics and clinical care. Similarly there will be need to overcome in vitro diagnostic regulatory hurdles with processes that allow accreditation of the combined use of steroid profiling and ML.

In terms of clinical implementation, numerous laboratories are now already moving away from LC-MS/MS-based unidimensional assays of single analytes to larger panels of many steroids.[26-36] Associated with these developments there are now already ongoing initiatives for harmonization of assay results across laboratories.[37] Large multicenter prospective studies and patient registries resulting from well-organized collaborative networks, such as the European Network for the Study of Adrenal Tumours (ENSAT),[38] can also facilitate collections of sufficient patient data and materials required for ML.

As far as integrating artificial with laboratory information management systems (LIMS), there have been initiatives underway for some time that make it possible to incorporate machine learning algorithms into LIMS.[39] In this way and as outlined above for the PROSALDO trial, reports may be generated from multidimensional diagnostic data to indicate probabilities of disease. With further developments and clarifications of disease prevalences among different populations it may also be possible for future fine-tuning of algorithms to produce patient-personalized post-test probabilities of disease based on know pre-test prevalences according to ethnicity and other predictive factors such as sex and age.

There are also already numerous examples of artificial intelligence and machine learning in diagnostics,[28,40-45] most related to imaging or pathological analyses of tissue specimens, but some also involving mass spectrometry-based applications.[28,43-45] The time is therefore right to starting considering how such approaches might be implemented within the clinical routine. For the subject of the present report, PA, the ultimate outcome might be improved identification and treatment of the current majority of patients in whom the disease remains unrecognized.

Sources of funding: This study was supported by the Deutsche Forschungsgemeinschaft (DFG, German Research Foundation Project number: 314061271-TRR 205/1 to FB, GE, JL, MR, TAW), the Else Kröner-Fresenius Stiftung (in support of the German Conns Registry-Else-Kröner Hyperaldosteronism Registry, 2013_A182 and 2015_A171), the European Research Council under the European Union’s Horizon 2020 research and innovation programme (grant agreement No [694913] to MR) and by funding dedicated to the Department of Medical Sciences from the Italian Ministry for Education, University and Research (Ministero dell'Istruzione, dell'Università e della Ricerca - MIUR) under the programme "Dipartimenti di Eccellenza 2018 – 2022", Progetto Strategico di Eccellenza Dipartimentale, DSM, UNITO - Project n. D15D18000410001.

eReferences

© 2020 Eisenhofer G et al. *JAMA Network Open.*

1. Eisenhofer G, Dekkers T, Peitzsch M, Dietz AS, Bidlingmaier M, Treitl M, et al. Mass Spectrometry-Based Adrenal and Peripheral Venous Steroid Profiling for Subtyping Primary Aldosteronism. Clin Chem 2016;62:514-24.
2. Williams TA, Peitzsch M, Dietz AS, Dekkers T, Bidlingmaier M, Riester A, et al. Genotype-Specific Steroid Profiles Associated With Aldosterone-Producing Adenomas. Hypertension 2016;67:139-45.
3. Funder JW, Carey RM, Mantero F, Murad MH, Reincke M, Shibata H, et al. The management of primary aldosteronism: case detection, diagnosis, and treatment: An Endocrine Society Clinical Practice Guideline. J Clin Endocrinol Metab 2016;101:1889-916.
4. Eisenhofer G, Peitzsch M, Kaden D, Langton K, Pamporaki C, Masjkur J, et al. Reference intervals for plasma concentrations of adrenal steroids measured by LC-MS/MS: Impact of gender, age, oral contraceptives, body mass index and blood pressure status. Clin Chim Acta 2017;470:115-24.
5. Williams TA, Lenders JWM, Mulatero P, Burrello J, Rottenkolber M, Adolf C, et al. Outcomes after adrenalectomy for unilateral primary aldosteronism: an international consensus on outcome measures and analysis of remission rates in an international cohort. Lancet Diabetes Endocrinol 2017;5:689-99.
6. Peitzsch M, Dekkers T, Haase M, Sweep FC, Quack I, Antoch G, et al. An LC-MS/MS method for steroid profiling during adrenal venous sampling for investigation of primary aldosteronism. J Steroid Biochem Mol Biol 2015;145:75-84.
7. Nyamundanda G, Poudel P, Patil Y, Sadanandam A. A Novel Statistical Method to Diagnose, Quantify and Correct Batch Effects in Genomic Studies. Sci Rep 2017;7:10849.
8. Zou H, Hastie T. Regularization and variable selection via the elastic net. . Journal of the Royal Statistical Society, 2005;67:301-20.
9. Breiman L. Random forests. Machine Learning 2001;45:5-32.
10. Meinshausen N. Quantile Regression Forests. Journal of Machine Learning Research 2006;7:983-99.
11. Allwein E, Schapire R, Singer Y. Reducing multiclass to binary: A unifying approach for margin classifiers. Journal of Machine Learning Research 2000;1:113-41.
12. Geladi P, Kowalski BR. Partial Least-Squares Regression: A Tutorial. Analytica Chimica Acta 1986;185:1-7.
13. Meyer LS, Wang X, Susnik E, Burrello J, Burrello A, Castellano I, et al. Immunohistopathology and Steroid Profiles Associated With Biochemical Outcomes After Adrenalectomy for Unilateral Primary Aldosteronism. Hypertension 2018;72:650-7.
14. Nanba K, Omata K, Else T, Beck PCC, Nanba AT, Turcu AF, et al. Targeted Molecular Characterization of Aldosterone-Producing Adenomas in White Americans. J Clin Endocrinol Metab 2018;103:3869-76.
15. Constantinescu G, Bidlingmaier M, Gruber M, Peitzsch M, Poitz DM, van Herwaarden AE, et al. Mass spectrometry reveals misdiagnosis of primary aldosteronism with scheduling for adrenalectomy due to immunoassay interference. Clin Chim Acta 2020;507:98-103.
16. Lenzini L, Rossitto G, Maiolino G, Letizia C, Funder JW, Rossi GP. A Meta-Analysis of Somatic KCNJ5 K(+) Channel Mutations In 1636 Patients With an Aldosterone-Producing Adenoma. J Clin Endocrinol Metab 2015;100:E1089-95.
17. Nanba K, Omata K, Gomez-Sanchez CE, Stratakis CA, Demidowich AP, Suzuki M, et al. Genetic Characteristics of Aldosterone-Producing Adenomas in Blacks. Hypertension 2019;73:885-92.
18. Funder JW. Primary Aldosteronism. Hypertension 2019;74:458-66.
19. Brown JM, Siddiqui M, Calhoun DA, Carey RM, Hopkins PN, Williams GH, Vaidya A. The Unrecognized Prevalence of Primary Aldosteronism. Ann Intern Med 2020.
20. Jansen PM, van den Born BJ, Frenkel WJ, de Bruijne EL, Deinum J, Kerstens MN, et al. Test characteristics of the aldosterone-to-renin ratio as a screening test for primary aldosteronism. J Hypertens 2014;32:115-26.
21. O'Shea PM, Griffin TP, Denieffe S, Fitzgibbon MC. The aldosterone to renin ratio in the diagnosis of primary aldosteronism: Promises and challenges. Int J Clin Pract 2019;73:e13353.
22. Guo Z, Poglitsch M, McWhinney BC, Ungerer JPJ, Ahmed AH, Gordon RD, et al. Measurement of Equilibrium Angiotensin II in the Diagnosis of Primary Aldosteronism. Clin Chem 2020;66:483-92.
23. Burrello J, Buffolo F, Domenig O, Tetti M, Pecori A, Monticone S, et al. Renin-Angiotensin-Aldosterone System Triple-A Analysis for the Screening of Primary Aldosteronism. Hypertension 2020;75:163-72.
24. Guo Z, Poglitsch M, Cowley D, Domenig O, McWhinney BC, Ungerer JPJ, et al. Effects of Ramipril on the Aldosterone/Renin Ratio and the Aldosterone/Angiotensin II Ratio in Patients With Primary Aldosteronism. Hypertension 2020:HYPERTENSIONAHA12014871.
25. Eisenhofer G, Duran C, Chavakis T, Cannistraci CV. Steroid metabolomics: machine learning and multidimensional diagnostics for adrenal cortical tumors, hyperplasias, and related disorders. Current Opinion in Endocrine and Metabolic Research 2019.
26. Kerkhofs TM, Kerstens MN, Kema IP, Willems TP, Haak HR. Diagnostic Value of Urinary Steroid Profiling in the Evaluation of Adrenal Tumors. Horm Cancer 2015;6:168-75.

© 2020 Eisenhofer G et al. *JAMA Network Open.*

27. Stolze BR, Gounden V, Gu J, Elliott EA, Masika LS, Abel BS, et al. An improved micro-method for the measurement of steroid profiles by APPI-LC-MS/MS and its use in assessing diurnal effects on steroid concentrations and optimizing the diagnosis and treatment of adrenal insufficiency and CAH. J Steroid Biochem Mol Biol 2015.
28. Arlt W, Biehl M, Taylor AE, Hahner S, Libe R, Hughes BA, et al. Urine steroid metabolomics as a biomarker tool for detecting malignancy in adrenal tumors. J Clin Endocrinol Metab 2011;96:3775-84.
29. Boelen A, Ruiter AF, Claahsen-van der Grinten HL, Endert E, Ackermans MT. Determination of a steroid profile in heel prick blood using LC-MS/MS. Bioanalysis 2016;8:375-84.
30. Deng Y, Zhang Y, Li S, Zhou W, Ye L, Wang L, et al. Steroid hormone profiling in obese and nonobese women with polycystic ovary syndrome. Sci Rep 2017;7:14156.
31. Hines JM, Bancos I, Bancos C, Singh RD, Avula AV, Young WF, et al. High-Resolution, Accurate-Mass (HRAM) Mass Spectrometry Urine Steroid Profiling in the Diagnosis of Adrenal Disorders. Clin Chem 2017.
32. Taylor DR, Ghataore L, Couchman L, Vincent RP, Whitelaw B, Lewis D, et al. A 13-Steroid Serum Panel Based on LC-MS/MS: Use in Detection of Adrenocortical Carcinoma. Clin Chem 2017.
33. Fanelli F, Di Dalmazi G. Serum steroid profiling by mass spectrometry in adrenocortical tumors: diagnostic implications. Curr Opin Endocrinol Diabetes Obes 2019;26:160-5.
34. Schweitzer S, Kunz M, Kurlbaum M, Vey J, Kendl S, Deutschbein T, et al. Plasma steroid metabolome profiling for the diagnosis of adrenocortical carcinoma. Eur J Endocrinol 2019;180:117-25.
35. Nanba AT, Rege J, Ren J, Auchus RJ, Rainey WE, Turcu AF. 11-Oxygenated C19 Steroids Do Not Decline With Age in Women. J Clin Endocrinol Metab 2019;104:2615-22.
36. Shafigullina ZR, Velikanova LI, Vorokhobina NV, Shustov SB, Lisitsin AA, Malevanaia EV, et al. Urinary steroid profiling by gas chromatography mass spectrometry: Early features of malignancy in patients with adrenal incidentalomas. Steroids 2018;135:31-5.
37. Greaves RF, Ho CS, Loh TP, Chai JH, Jolly L, Graham P, et al. Current state and recommendations for harmonization of serum/plasma 17-hydroxyprogesterone mass spectrometry methods. Clin Chem Lab Med 2018;56:1685-97.
38. Stell A, Sinnott R. The ENSAT registry: a digital repository supporting adrenal cancer research. Stud Health Technol Inform 2012;178:207-12.
39. Calva D, Lehman M. An analysis of the possible applications of artificial intelligence techniques to a clinical laboratory information management system. International Journal of Computer Science and Network Security 2008;12:82-6.
40. Guncar G, Kukar M, Notar M, Brvar M, Cernelc P, Notar M, Notar M. An application of machine learning to haematological diagnosis. Sci Rep 2018;8:411.
41. Komura D, Ishikawa S. Machine learning approaches for pathologic diagnosis. Virchows Arch 2019;475:131-8.
42. Nichols JA, Herbert Chan HW, Baker MAB. Machine learning: applications of artificial intelligence to imaging and diagnosis. Biophys Rev 2019;11:111-8.
43. Wilkes EH, Rumsby G, Woodward GM. Using Machine Learning to Aid the Interpretation of Urine Steroid Profiles. Clin Chem 2018;64:1586-95.
44. Subhashini P, Jaya Krishna S, Usha Rani G, Sushma Chander N, Maheshwar Reddy G, Naushad SM. Application of machine learning algorithms for the differential diagnosis of peroxisomal disorders. J Biochem 2019;165:67-73.
45. Sans M, Zhang J, Lin JQ, Feider CL, Giese N, Breen MT, et al. Performance of the MasSpec Pen for Rapid Diagnosis of Ovarian Cancer. Clin Chem 2019;65:674-83.

© 2020 Eisenhofer G et al. *JAMA Network Open.*
